# Analyses of GWAS and Sub‐Threshold Loci Lead to the Discovery of Dendrite Development and Morphology Dysfunction Underlying Schizophrenia Genetic Risk

**DOI:** 10.1002/advs.202508519

**Published:** 2025-08-29

**Authors:** Rui Chen, Benjamin Siciliano, Quan Wang, Chongchong Xu, Qiang Wei, Hai Yang, James S Sutcliffe, Yi Jiang, Ying Ji, Chunyu Liu, Feixiong Cheng, Edwin H Cook, Nancy J Cox, Xue Zhong, Zhexing Wen, Bingshan Li

**Affiliations:** ^1^ Department of Molecular Physiology and Biophysics Vanderbilt University Nashville TN 37232 USA; ^2^ Vanderbilt Genetics Institute Vanderbilt University Medical Center Nashville TN 37232 USA; ^3^ The Graduate Program in Molecular and Systems Pharmacology Laney Graduate School Emory University Atlanta GA 30322 USA; ^4^ Department of Psychiatry and Behavioral Sciences Emory University School of Medicine Atlanta GA 30322 USA; ^5^ Department of Psychiatry SUNY Upstate Medical University Syracuse NY 13210 USA; ^6^ Department of Molecular Medicine Cleveland Clinic Lerner College of Medicine Case Western Reserve University Cleveland OH 44106 USA; ^7^ Cleveland Clinic Genome Center and Genomic Medicine Institute Lerner Research Institute Cleveland Clinic Cleveland OH 44106 USA; ^8^ Department of Psychiatry University of Illinois Chicago IL 60612 USA; ^9^ Department of Medicine Division of Genetic Medicine Vanderbilt University Medical Center Nashville TN 37232 USA; ^10^ Department of Cell Biology Emory University School of Medicine Atlanta GA 30322 USA; ^11^ Department of Neurology Emory University School of Medicine Atlanta GA 30322 USA; ^12^ Department of Human Genetics Emory University School of Medicine Atlanta GA 30322 USA

**Keywords:** dendrite development, genetic risk, GWAS, neurodevelopmental, psychiatric disorder, Schizophrenia, sub‐threshold loci

## Abstract

Schizophrenia (SCZ) is highly polygenic, and its biological underpinnings remain unclear. In this study, a cost‐effective strategy of including sub‐threshold GWAS (subGWAS) loci (i.e., 5 × 10^−8^ < *P* ≤ 10^−6^) in analysis is explored to increase the inference power of novel pathways. A total of 180 subGWAS loci are identified from SCZ GWAS studies and are shown to contain substantial true genetic association signals. By jointly modeling GWAS (sigGWAS) and subGWAS loci, 304 high‐confidence risk genes (HRGs) are identified, as well as a novel category of biological processes detected only in subGWAS loci, *i.e., dendrite development and morphogenesis* (DDM). Two candidate DDM genes (*CUL7* and *DCC*), whose risk alleles in GWAS are associated with increased expression, are examined, and it is observed that upregulation of these genes leads to reduced neurite length. It is further revealed that the DDM genes lead to disrupted regulatory programs of the transcription factors *CUX1/2 and NEUROD1*. Collectively, the study identifies DDM as a novel biological process in SCZ susceptibility, with particular implications for *DCC*‐ and *CUL7*‐mediated alterations in neurite development and reveals regulatory programs involved in perturbation of the two candidate genes.

## Graphical abstract

A cost‐effective strategy is developed analyzing sub‐threshold GWAS loci (5 × 10^−8^ < P ≤ 10^−6^), identifying 180 risk loci and 304 high‐confidence genes through combined GWAS/subGWAS analysis. This approach reveals dendrite development and morphogenesis (DDM) as a novel SCZ pathway. Functional validation shows DDM genes CUL7 and DCC impair neurite growth and disrupt neural regulators (CUX1/2, NEUROD1), uncovering new SCZ mechanisms.

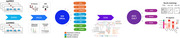

## 1 Introduction

Schizophrenia (SCZ), a psychiatric disorder afflicting approximately 1% of the population, is a leading cause of disability and premature death.^[^ ^1^, ^2^ ^]^ SCZ is highly heritable, with an estimated heritability of ∼80%.^[^ ^3^, ^4^ ^]^ Incomplete understanding of disease pathophysiology continues to hinder the development of new therapeutics beyond the advent of antipsychotic medications 50 years ago.^[^ ^5^ ^]^ Identifying risk genes is key to facilitating drug development.^[^ ^6^ ^]^ During the past decade, more than two hundred loci associated with SCZ have been identified from Genome‐wide association studies (GWAS).^[^ ^7^, ^8^, ^9^ ^]^ However, the discovered loci collectively explain only a small proportion of the heritability, suggesting that the vast majority of risk loci have yet to be discovered. Risk genes have been implicated based on GWAS loci,^[^ ^10^ ^]^ however, given that thousands of genes are likely involved in SCZ risk,^[^ ^11^ ^]^ discoveries are constrained by the limited number of known loci, hindering the discovery of novel mechanisms. In parallel to increasing GWAS samples, alternative approaches to make novel discoveries are viable. Several studies have shown that GWAS loci slightly below the genome‐wide significance threshold (*P* = 5 × 10^−8^) account for most of the “missing heritability” in GWAS;^[^ ^12^, ^13^ ^]^ we refer to loci with ‘sub‐threshold’ significance as subGWAS loci hereinafter. The widely accepted genome‐wide significance threshold for GWAS is based on a conservative Bonferroni correction, and adoption of such a stringent threshold possibly neglects numerous subGWAS loci that may reach genome‐wide significance by inclusion of additional data.^[^ ^14^ ^]^ A study implicated that subGWAS loci could harbor plausible functional, disease‐relevant signals.^[^ ^15^, ^16^ ^]^ These findings suggest that exploration into subGWAS loci has real potential to make further discoveries, given the current wealth of data.

Despite the success of identifying risk loci through GWAS, pinpointing corresponding risk genes remains a major obstacle to map variants to functions, since most GWAS loci are located in the noncoding regions of the genome.^[^ ^17^ ^]^ Several gene sets and pathways relevant to SCZ pathology have been proposed.^[^ ^8^, ^18^, ^19^ ^]^ For instance, the involvement of synaptic processes has been repeatedly reported,^[^ ^20^, ^21^, ^22^, ^23^, ^24^ ^]^ and this can explain a progressive cortical grey matter reduction observed in imaging studies.^[^ ^25^, ^26^, ^27^ ^]^ In spite of these findings, our knowledge of the disease pathophysiology remains limited. Expanding analysis to include subGWAS loci, coupled with rigorous methodology, has the potential to identify bona fide risk loci and effectively increase our ability to reveal novel insights into SCZ mechanisms.

In this study, we took advantage of the values of subGWAS loci (5 × 10^−8^ < *P* ≤ 1 × 10^−6^) in SCZ,^[^ ^8^ ^]^ and combined subGWAS and genome‐wide significant GWAS (sigGWAS) loci to make robust mechanistic inferences based on the expanded set of loci. Specifically, we chose the Integrative Risk Gene Selector (iRIGS), an integrative Bayesian framework to nominate risk genes at GWAS loci,^[^ ^10^ ^]^ to jointly analyze both sigGWAS and subGWAS loci to predict risk genes, leveraging the method's robust accuracy by integrating multi‐level genomic data. After rigorous validation from multiple orthogonal angles, we identified a novel category of biological processes involved in neuronal *dendrite development and morphogenesis* (DDM), key functions that are essential for normal synapse formation and maturation. Notably, these biological processes are only enriched in subGWAS loci while combining with sigGWAS loci, supporting the value of subGWAS in identifying novel pathways with weaker effect sizes that are otherwise hard to discover without explicitly analyzing subGWAS loci. We validated the role of DDM in SCZ from multiple perspectives and replicated its involvement in other psychiatric disorders, including bipolar disorder (BD) and major depressive disorder (MDD), supporting the robustness of the novel pathways.

We further explored the function of the DDM pathways in human induced pluripotent stem cell (hiPSC)‐derived neurons. Specifically, we selected two candidate risk genes in subGWAS loci, i.e., *CUL7* and *DCC*, based on spatial and temporal expression patterns as well as brain tissue expression quantitative trait loci (eQTL) support. We tested the impact of the genes on neuronal morphogenesis as well as on transcriptomic reprogramming by manipulating their expressions in human neurons. We observed that upregulation of these genes resulted in abnormal dendrite development, namely significantly reduced neurite lengths compared to controls, and transcriptomic changes in genes associated with neuronal development pathways. Furthermore, transcriptional regulatory network analysis identified disrupted regulons involving transcription factors *CUX1/2*, *NEUROG1*, etc.

## 2 Results

### 2.1 SCZ subGWAS Loci in Large‐Scale GWAS Contain Substantial True Associated Variants

To investigate whether subGWAS loci harbor valid signals, we analyzed three largest SCZ GWAS data with varying sample sizes,^[^ ^7^, ^8^, ^9^ ^]^ referred as PGC (36,989 cases and 113,075 controls), CLOZUK+PGC (40,675 cases and 64,643 controls) and PGC3 (76,755 cases and 243,649 controls) hereinafter, examining whether subGWAS loci could achieve genome‐wide significance with increased sample sizes. While PGC and identified 108 loci, CLOZUK+PGC detected 145 sigGWAS loci, including 52 new loci not found in PGC. We assessed whether these new loci overlap with the subGWAS loci from the PGC by analyzing linkage disequilibrium (LD). At a stringent threshold of *r* ^2^ ≥ 0.9, 21 of 38 (55%) the new loci overlap with subGWAS loci in PGC, while a lenient criterion (*r* ^2^ ≥ 0.2) revealed overlap for 28 of 52 (54%) loci (Figure S1A, Supporting Information). Variants within these 52 loci showed improved significance in CLOZUK+PGC (e.g., Figure S2, Supporting Information). This trend remains when comparing the CLOZUK+PGC with the largest GWAS, PGC3 (Figure S1B, Supporting Information).^[^ ^9^ ^]^ Altogether, these results show that subGWAS loci in SCZ GWAS contain true genetic signals.

### 2.2 SCZ subGWAS Loci Are Enriched in Brain Tissue Enhancers

Most GWAS loci reside in non‐coding regions, and thus affect their target genes through regulatory effects.^[^ ^28^ ^]^ To assess the functional relevance of subGWAS loci in the brain, we examined their overlap with brain enhancers (Methods). We observed significantly more subGWAS loci's LD regions (subGWAS LD regions) overlapped with dorsolateral prefrontal cortex (DLPFC) enhancers (132 vs 107; *P* < 1 × 10^−3^; Figure S3A, Supporting Information; Methods), and each subGWAS LD region overlaps significantly more enhancers on average (6.54 vs 5.16; *P* = 5 × 10^−3^; Figure S3B, Supporting Information), a pattern replicated in PGC3 (196 versus 102 and 7.2 versus 5.17, Figure S3B, Supporting Information). Tissue‐specific analysis demonstrated greater subGWAS enhancers in brain tissues, especially DLPFC, compared to non‐brain tissues (*P* = 7.5 × 10 ^−5^; Figure S4A, Supporting Information). These patterns of significance also hold for sigGWAS LD regions and the largest GWAS PGC3 (Figures S3A and S4A,B, Supporting Information).

### 2.3 Risk Genes Inferred from SCZ subGWAS Harbor Genuine SCZ Risk Genes

To identify high‐confidence risk genes (HRGs), we applied iRIGS,^[^ ^10^ ^]^ a Bayesian framework that integrates GWAS data, multi‐omics features (including regulatory interactions and de novo mutations), and gene network information. This method evaluates candidate genes within ±1 Mb of each GWAS locus's index SNP and probabilistically prioritizes genes for each locus. The gene at a locus with the highest posterior probability (PP) is selected as the HRG of the corresponding locus. The PP is calculated in a Bayesian framework based on multi‐omics data and gene interaction networks, with the rationale that risk genes often have multi‐level supportive data and tend to converge to a few core gene modules. Additional details are in Methods. We mainly focused on the GWAS of CLOZUK+PGC to prove our concept and used PGC3 as a validation dataset to ensure our discoveries are robust. We combined subGWAS and sigGWAS loci together and identified 304 HRGs (allHRGs), comprising 135 (sigHRGs) and 177 (subHRGs) from sigGWAS and subGWAS loci, respectively (Methods; Table S1, Supporting Information). The iRIGS also identified local background genes (706 sigLBGs, 862 subLBGs, and 1517 allLBGs) as more appropriate controls than whole genome background genes.

Using stratified LD score regression (LDSC) method^[^ ^29^ ^]^ (Methods), we observed noticeably increased enrichment of heritability in subHRGs (enrichment  =  8.97, *P*  =  1.51  ×  10^−4^) compared to subLBGs (enrichment = 3.38, *P* = 2.28 × 10^−3^, Figures S5 and S6, Supporting Information).

Exploration of spatiotemporal expression pattern (GTEx^[^ ^30^ ^]^ and Brainspan^[^ ^31^ ^]^) revealed strong brain specificity for sigHRGs (*P* = 8.21 × 10^−7^; one‐sided Wilcoxon test) and reduced but obvious brain specificity for subHRGs (*P* = 2.58 × 10^−3^), compared to LBGs (Figure S7, Supporting Information). In addition, we also observed higher expression levels of both sigHRGs (*P* = 4.37 × 10^−4^) and subHRGs (*P* = 4.48 × 10^−4^, Figure S8, Supporting Information) at prenatal stages compared to postnatal stages. These results are consistent with the previously reported spatiotemporal expression pattern of SCZ risk genes.^[^ ^10^, ^32^ ^]^

### 2.4 subHRGs Inferred from subGWAS Loci Expand the Knowledge Discovered Only by sigHRGs

To determine whether subGWAS provides complementary biological insights beyond sigGWAS loci, we performed gene set enrichment analysis (GSEA) on 18 functional gene sets previously implicated in SCZ (Methods). Notably, subGWAS loci drive stronger enrichment significance in the 7 gene sets already significant in sigGWAS, e.g., evolutionarily constrained genes (ECG) and miRNA‐137 (miR‐137) targets (**Table** **1**). Importantly, combining subHRGs and sigHRGs led to new significant gene sets that neither group alone identified separately, e.g., the presynaptic active zone (PRAZ) (*P*_all_ = 6.22 × 10^−3^), calcium channel and signaling (CCS, *P_all_* = 3.55 × 10^−2^), and metabotropic glutamate receptor subtype 5 (mGluR5, *P_all_* = 1.38 × 10^−2^) sets. More interestingly, subHRGs alone detected additional relevent gene sets, e.g., fragile X mental retardation protein (FMRP) targets identified by Ascano et al (2012) (*P*_sub_ = 7.39 × 10^−3^, *P*_sig_ = 0.23).

**Table 1:** GSEA with previously implicated SCZ‐related gene sets.

Gene set^a)^	allHRGs		sigHRGs		subHRGs	
	*P*_correction_	OR^b)^	*P*_correction_	OR	*P*_correction_	OR
ARC (25)	1	5.01 (2/4)	1	Inf (1/1)	1	2.44 (1/3)
AutDB (781)	5.39 × 10^−21^	8.77 (56/94)	4.19 × 10^−13^	10.72 (31/50)	2.07 × 10^−7^	5.98 (25/48)
CCS (73)	3.55 × 10^−2^	12.64 (5/7)	5.03 × 10^−2^	21.4 (4/5)	1	4.88 (1/2)
ECG (998)	8.76 × 10^−17^	7.25 (50/90)	6.50 × 10^−11^	7.77 (31/57)	1.65 × 10^−6^	6.29 (21/39)
Essential genes (3910)	1.85 × 10^−15^	3.16 (121/383)	9.12 × 10^−7^	3.06 (54/180)	5.13 × 10^−10^	3.4 (70/209)
FMRP‐Ascano (939)	2.67 × 10^−4^	2.81 (32/93)	0.234	2.28 (14/48)	7.39 × 10^−3^	3.13 (18/48)
FMRP‐Darnel (832)	1.21 × 10^−10^	4.83 (43/93)	1.01 × 10^−5^	4.78 (23/52)	1.76 × 10^−4^	4.26 (20/45)
GABA (18)	1	5 (1/2)	1	0 (0/1)	1	Inf (1/1)
mGluR5 (37)	1.38 × 10^−2^	Inf (4/4)	7.31 × 10^−2^	Inf (3/3)	1	Inf (1/1)
miR‐137 targets (281)	3.04 × 10^−6^	6.66 (19/34)	2.11 × 10^−3^	8.3 (9/15)	1.12 × 10^−2^	5.09 (10/20)
NMDAR (59)	1	10.02 (2/3)	1	5.25 (1/2)	1	Inf (1/1)
PRAZ (209)	6.22 × 10^−3^	6.57 (9/16)	0.27	7.13 (4/7)	0.288	4.97 (5/10)
PRP (336)	0.057	3.65 (10/24)	1	2.97 (5/14)	0.468	4.14 (5/11)
PSD (1444)	7.39 × 10^−5^	2.74 (38/113)	9.06 × 10^−3^	2.96 (19/56)	4.13 × 10^−2^	2.42 (20/63)
PSD‐95 (107)	0.558	5.04 (4/8)	0.252	15.94 (3/4)	1	3.27 (2/5)
RBFOX1 (556)	2.27 × 10^−6^	5.31 (23/46)	5.1 × 10^−4^	6.77 (12/22)	1.52 × 10^−2^	4.32 (11/24)
SYV (107)	1	2 (2/7))	1	0 (0/3)	1	4.9 (2/4)
TADA (179)	0.198	3.39 (8/20)	0.234	5.38 (5/10)	1	2.47 (4/12)

^a)^ The numbers of genes in the corresponding gene sets are in parentheses;

^b)^ The numbers of genes in parentheses stand for the overlap gene numbers in HRG/(HRG+LPG). One‐sided Fisher's exact test and Bonferroni correction were used for enrichment analyses. Significance was defined as P ≤ 0.05. Please refer to the Methods for details of gene set abbreviations. OR: odds ratio. Red: Enhanced p values from combining sigGWAS and subGWAS; Blue: New significant gene sets from combining sigGWAS and subGWAS; Yellow: New significant gene sets from only subGWAS.

**Figure 1:**
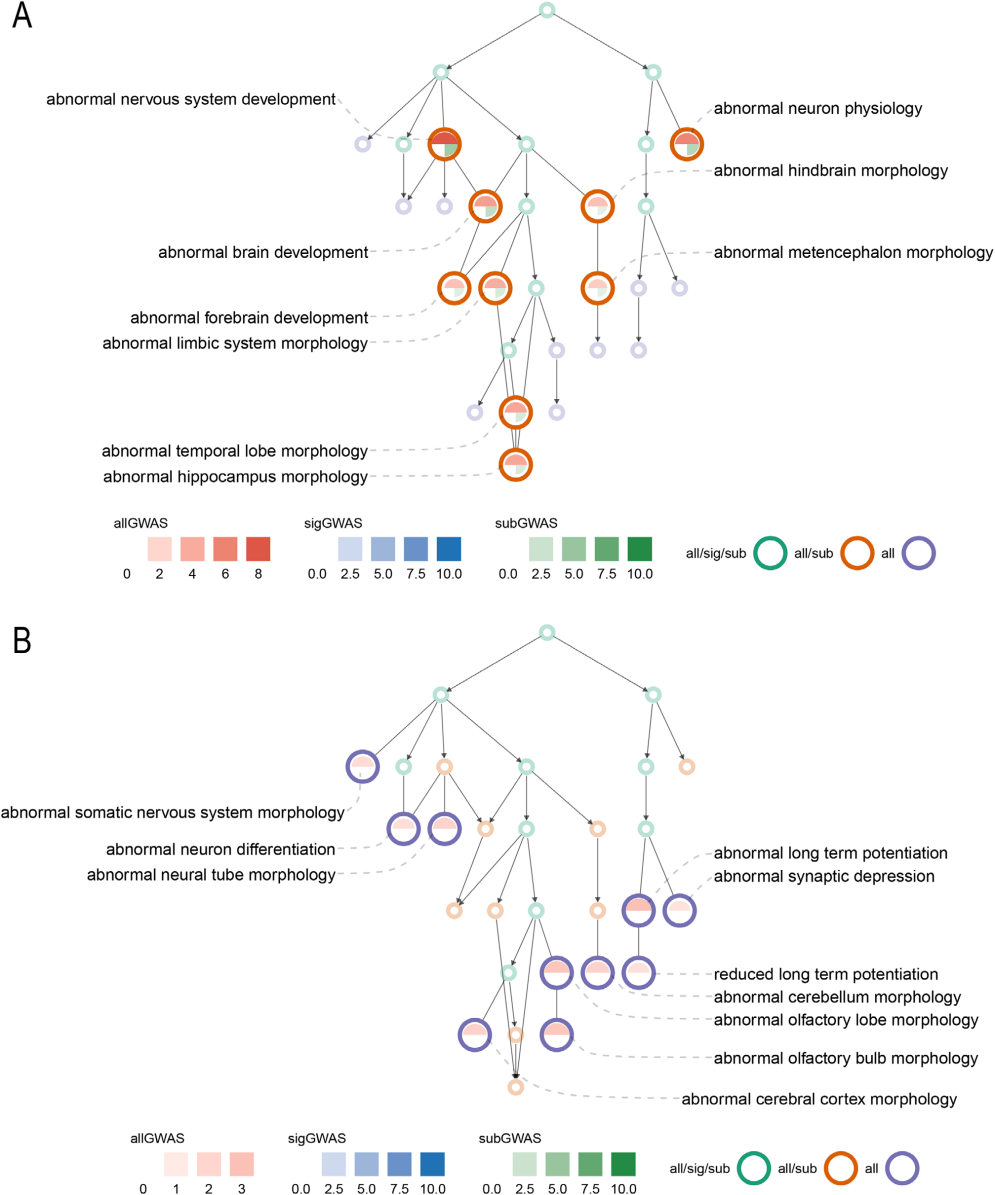
Directed acyclic graphs of MPO terms under nervous system phenotypes. (A) Terms identified by subGWAS independently. (B) Terms identified only by a combination of subGWAS and sigGWAS, indicating the boosted power of subGWAS. all/sig/sub (green): significant in allHRGs, sigHRGs, and subHRGs; all/sub (orange): significant in allHRGs and subHRGs; all (blue): significant in allHRGs. The terminology is adopted by other DAGs as well. The −log_10_(*P‐*value) of enrichment is represented by gradient colors, which is applied to other DAGs if not otherwise specified. P values are derived from the Gene Set Enrichment Analysis (GSEA) with MPO/GO terms using two‐sided Fisher's exact tests and following by Q‐value correction. Significance was defined as P ≤ 0.05 unless noted.

We also investigated phenotypic manifestations in mouse gene knockout data (Methods). We observed that 201 of 1721 terms from the Mammalian Phenotype Ontology (MPO) were significantly enriched in allHRGs (Table S2, Supporting Information). First, the *nervous system phenotype* branch (*P* = 6.2 × 10^−21^) stands out from these enriched terms (Figure S9, Supporting Information), consistent with SCZ as a neurodevelopmental disorder.^[^ ^33^ ^]^ Second, 40 terms are exclusively identified by subHRGs, e.g., *abnormal hindbrain morphology* (*P*_sub_ = 4.82 × 10^−2^, *P*_sig_ = 1) and *abnormal brain development* (*P*_sub_ = 1.13 × 10^−3^, *P*_sig_ = 1) (**Figure** **1**A). Third, 80 terms that sigHRGs and subHRGs failed to identify individually (Figure 1B), including *abnormal synaptic depression* (*P*_all_ = 4.03 × 10^−2^) and *abnormal neuron differentiation* (*P*_all_ = 2.46 × 10^−2^). These findings are consistent with observations from GSEA with gene sets known to be implicated in SCZ, showing that subGWAS loci are valuable to augment our understanding of SCZ disease etiology.

### 2.5 SCZ subHRGs Lead to Novel Discovery of Dendrite Development and Morphogenesis Implicated in SCZ Genetic Risk

**Figure 2:**
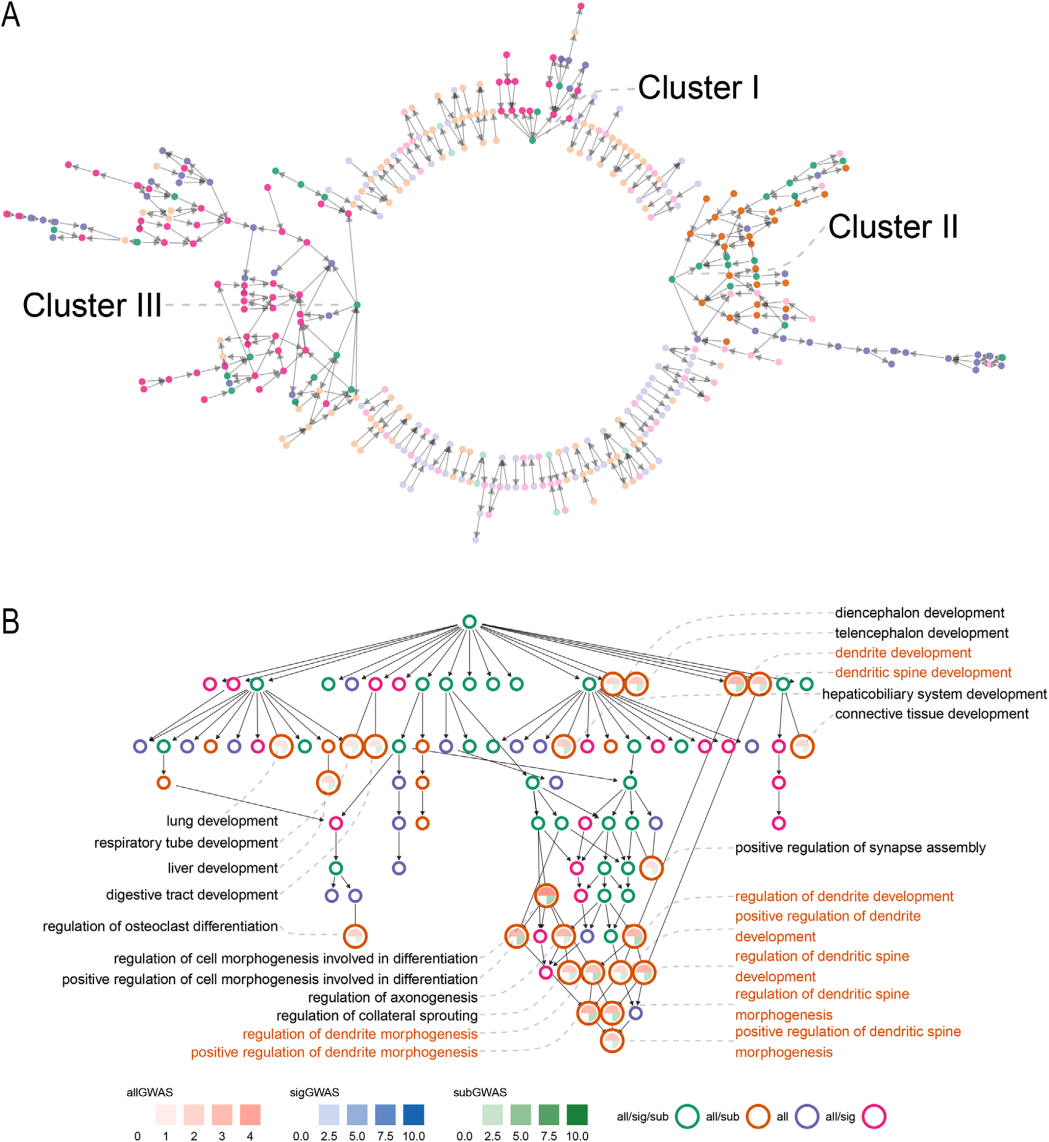
GSEA enrichment in GO terms. (A) DAG of GSEA enrichment in GO terms with genes ≤ 500. Three clusters of terms are identified: Cluster I and III are mostly contributed by all/sig and all/sig/sub and Cluster II is mostly contributed by all/sub and all/sig/sub. (B) DAG of dendrite related terms under anatomical structure development (GO:0048856) identified by subGWAS. Dendrite terms are marked in orange texts.

To gain more specific functional insights into SCZ pathophysiology, we performed GSEA with Gene Ontology (GO) (Methods), which revealed significantly enriched terms across different layers of the hierarchical GO structure (Figure S10 and Table S3, Supporting Information). Focusing on terms of ≤ 500 genes for more specific interpretation (**Figure** **2**A), we identified three major clusters. SigHRGs make the major contribution to the identification of both clusters I and III (Figure 2A, pink dots), in which enriched GO terms are related to well‐known implicated functions, including synapse related processes^[^ ^23^ ^]^ in cluster I (Figure S11, Supporting Information) and ion transport channels (Giegling *et al.*, 2010; Berridge, 2013; Schizophrenia Working Group of the Psychiatric Genomics Consortium, 2014) in cluster III (Figure S12, Supporting Information).

In contrast, cluster II revealed a novel functional module involved in DDM predominantly identified by subHRGs (Figure 2A, orange dots), with specific enriched terms include *dendrite development* (DD, *P*_sub_ = 2.03 × 10^−3^, P_sig_ = 0.24), *dendrite morphogenesis* (DM, *P*_sub_ = 1.67 × 10^−3^, *P*_sig_ = 0.24), *regulation of dendrite development* (RDD, *P*_sub_ = 4.86 × 10^−4^, *P*_sig_ = 0.4), and *regulation of dendrite morphogenesis* (RDM, *P*_sub_ = 7.61 × 10^−4^, P_sig_ = 0.54) (Figure 2B large orage circles). DD term includes 10 genes overlapping subHRGs versus 4 genes overlapping with sigHRGs (Table S1, Supporting Information), which explains why subHRGs are the main driver in identifying this enriched module (Figure 2B; Table S1, Supporting Information). We also found that the enrichment mainly derived from overlaps between terms involving regulation function in subHRGs, i.e., 9 of 10 genes in common between DD and subHRGs derive from RDD (Table S1, Supporting Information). Literature evidence supports the involvement of DDM in SCZ (Table S4, Supporting Information). Other interesting terms identified by subGWAS include *neuron migration*, *glial cell migration* and others (Figures S13 and S14, Supporting Information).

### 2.6 Genes in Dendrite Development and Morphogenesis Terms Contribute to Heritability of SCZ and Other Relevant Psychiatric Disorders

**Figure 3:**
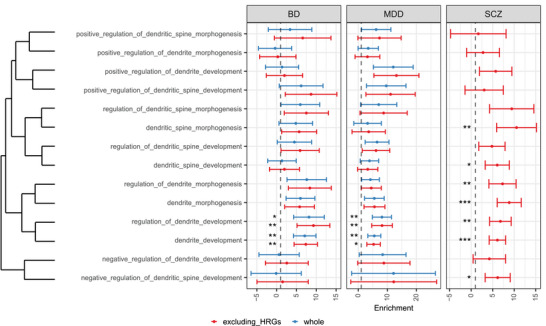
Dendrite‐related terms explained significant genetic risk of psychiatric disorders. The similarities of GO terms are computed using the Jaccard index method. The center values represent the enrichment, and the error bars indicate standard errors. The statistical method is adopted by LDSC method. *: P ≤ 0.1; **: P ≤ 0.05; ***: P ≤ 0.01.

To validate that DDM terms are genetically involved in SCZ, we conducted LDSC analysis using these terms while excluding allHRGs to avoid confounding effects from prior GWAS discoveries. We observed significant enrichment of heritability in DDM related terms (**Figure** **3**; *P*_DD_ = 8.25 × 10^−3^, *P_DM_* = 5.34 × 10^−3^). Extending analysis to related psychiatric disorders, bipolar disorder (20,352 cases and 31,358 controls)^[^ ^34^ ^]^ and major depressive disorder (245,363 cases and 561,190 controls),^[^ ^35^ ^]^ both of which share genetic susceptibility with SCZ,^[^ ^36^, ^37^ ^]^ we observed a similar enrichment of dendrite related terms in genetic risk of BD and MDD (Figure 3).

### 2.7 DDM Genes Are Enriched with Rare Coding Variants in SCZ and Show Expression Specificity in Neurons at Both Fetal and Adult Stages

Rare gene‐disrupting coding variants are more abundant in SCZ,^[^ ^38^ ^]^ and the affected genes likely overlap with common variants (mostly noncoding).^[^ ^7^ ^]^ Using rare variant association summary statistics data at gene level from SCHEMA (https://schema.broadinstitute.org/),^[^ ^39^ ^]^ we observed that DD and DM genes have significantly smaller *P*‐values than random background genes (*P_DD_* = 8.55 × 10^−6^, *P_DM_* = 1.62 × 10^−4^; Figure S15, Supporting Information).

**Figure 4:**
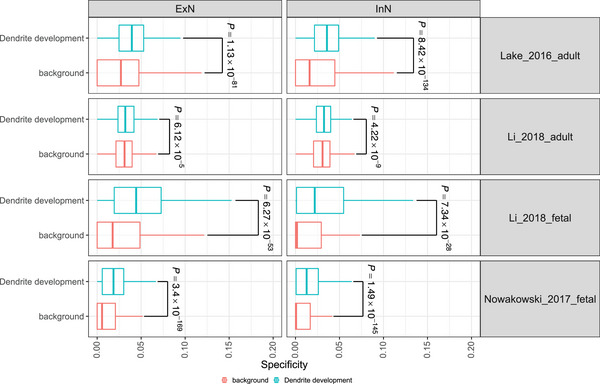
Expressions of dendrite development genes in neurons of fetal and adult brain. Dendrite related genes show higher expression than background in both fetal and adult brain, implying potential dendritic defects in both types of neurons at fetal and adult stages. The Wilcoxon test is used if not specified. The box plots show the median and the 25th and 75th percentiles. The whiskers extend from the box to the largest and smallest values no further than 1.5 times the interquartile range (IQR) from the box (or the distance between the 25th and 75th percentiles).

Current neuropathology models of SCZ implicate neurodevelopmental abnormalities spanning both fetal and postnatal stages,^[^ ^40^ ^]^ mainly supported by neuroimaging studies of adult brain.^[^ ^41^, ^42^, ^43^, ^44^ ^]^ Through analysis of four independent single‐cell/nucleus RNA‐seq datasets spanning fetal and adult brains,^[^ ^45^, ^46^, ^47^ ^]^ we observed that DD genes show high cell type specificity in both fetal and adult neurons compared to background (**Figure** **4**), including both excitatory (ExN) and inhibitory (InN) neurons. These results imply potential dendritic defects in both types of neurons at fetal and adult stages, supporting the revised neurodevelopment hypothesis of SCZ at molecular level.

### 2.8 Validation of CUL7 and DCC Genes in Dendrite Development in hiPSC‐Derived Neurons

**Figure 5:**
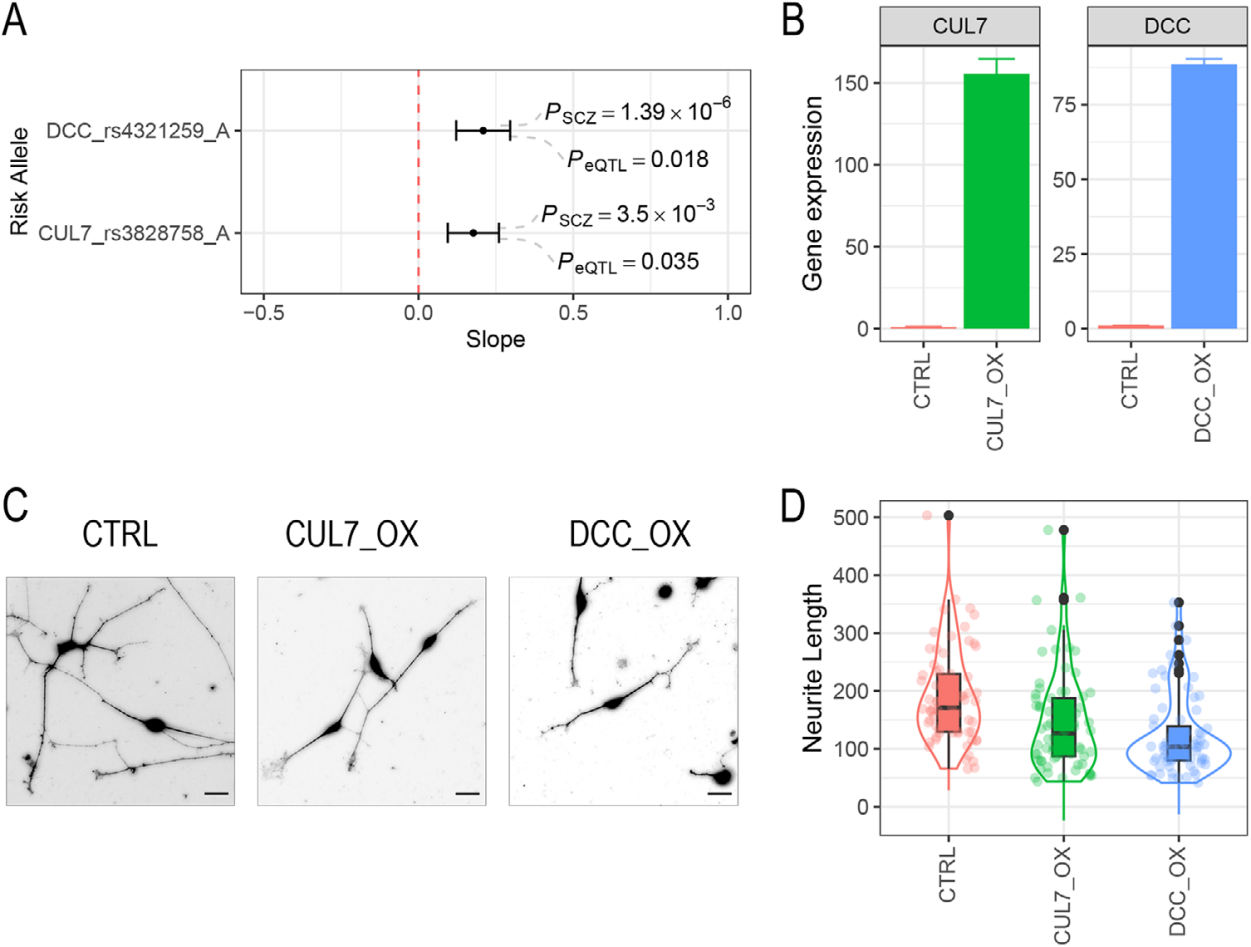
Overexpression of candidate genes (*CUL7* and *DCC*) in hiPSC‐derived neurons results in neurite deficit. (A) The slope of risk alleles for the two genes in fetal brain eQTL data. The positive slopes indicate upregulation of expression by risk alleles. (B) The results of RT‐qPCR after manipulation. The target genes are all overexpressed as expected. (C) Morphology of neurites of CUL7_OX and DCC_OX versus a control line. (D) Neurite lengths for CUL7_OX (N = 72) and DCC_OX (N = 87) are all significantly reduced compared to CTRL (N = 70) (p < 2.2 × 10‐16 for both genes; one‐sided t‐test).

To validate dendrite‐related risk genes in SCZ pathogenesis, we chose *DCC and CUL7*, based on multiple lines of evidence, including brain eQTL, specificity of fetal brain during development, and others. (Table S6, Supporting Information). To mimic the effects of risk genes in SCZ implicated by eQTL studies (**Figure** **5**A), we overexpressed these two genes in hiPSC‐derived neurons at day 4 after differentiation using lentiviral system (Methods; Figure 5B).

At day 10 of differentiation, morphological analysis revealed that dendrite development is significantly impaired (Figure 5C,D). Specifically, the *DCC* line showed 35% of neurite length reduction compared to the control (122.6 vs 189.66; P < 2.2 × 10^−16^; t‐test; Figure 5C), while the *CUL7* line demonstrated 24% shorter than the control (144.88 ves 189.66; *P* < 2.2 × 10^−16^; t‐test; Figure 5D).

### 2.9 Manipulation of CUL7 and DCC Led to Dysregulated Transcriptional Programs Associated With SCZ Risk

We conducted RNA‐seq on hiPSC‐derived neurons at day 10 (6 days after the genetic manipulations; Figure S16, Supporting Information), and revealed 1837 (DE_CUL7) and 2346 (DE_DCC) differentially expressed (DE) genes (fold change > 1.1 and FDR < 0.05; Table S7, Supporting Information; Methods). As the enrichment signals from gene sets with prior knowledge mainly come from down‐regulated DE genes (Table S8, Supporting Information), we focus on the down‐regulated DE genes in the analysis to follow (Table S9, Supporting Information).

**Figure 6:**
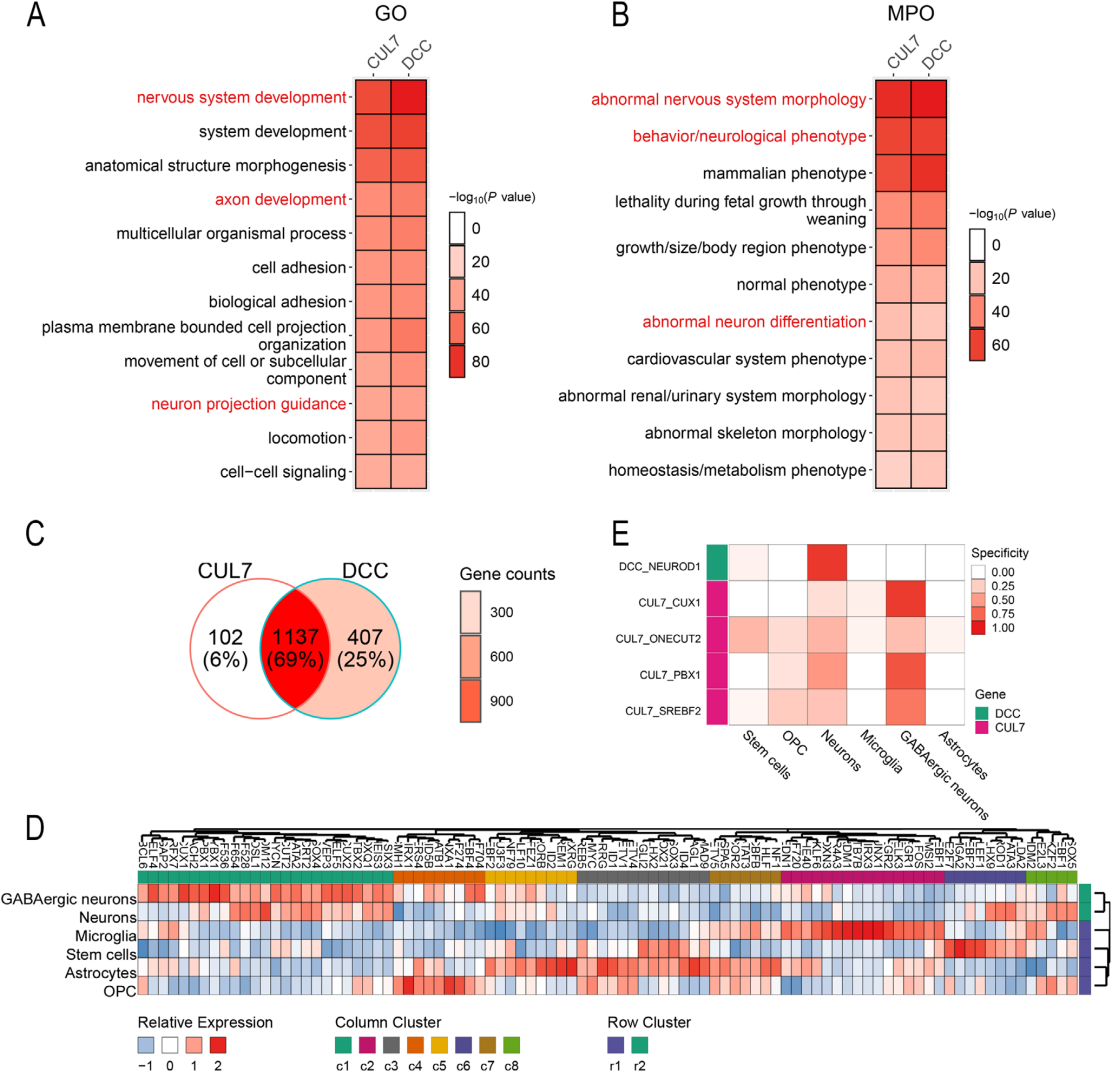
GSEA and transcriptional network analysis of DE genes from the two overexpression lines. Top 15 enriched GO (A) and MPO (B) clusters for two DE gene lists. Red color terms: neuron related term clusters. (C) Venn diagram of the two DE gene lists. (D) Expression of disturbed TFs in developmental brain. (E) The regulons identified from the two DE gene lists show specific activity in neurons.

DE_CUL7 is enriched functional gene sets (e.g. FRMP, PSD) (Table S8, Supporting Information) and neuron‐related GO clusters (Figure S17, Supporting Information), including *nervous system development* (*P* = 1.35 × 10^−73^) and *axon development* (*P* = 6.05 × 10^−49^; **Figure** **6**A). MPO analysis revealed enrichments in the clusters of *abnormal nervous system morphology* (*P* = 1.06 × 10^−64^) and *abnormal neuron differentiation* (*P* = 1.98 × 10^−22^) (Figure 6B). DE_CUL7 exhibited significant brain specificity (*P* <10^−16^) and upregulated expression during neuronal differentiation and fetal stages of brain development (Figure S19‐23). DE_CUL7 genes also demonstrate genetic evidence, including enrichments for SCZ heritability (*P* = 2.58 × 10^−6^). Rare coding variants (*P* = 4.57 × 10^−8^) and *de novo* mutations in developmental disorders (*P* = 3.5 × 10^−2^, Figure S23; Table S10, Supporting Information). DE_DCC genes show trends similar to DE_CUL7 (Tables S8–S10, and Figures S17 and S18, Supporting Information).

When comparing the overlapping genes among the two DE gene lists, we found that about 66% (1137) of genes are shared by DE_CUL7 and DE_DCC (Figure 6C). These genes show enrichment with more neuron‐related term clusters, indicating *CUL7* and *DCC* may be involved in the same functions regulating neurodevelopment (Figure S24A,B, Supporting Information).

Next, we sought to document transcriptional regulatory networks affected by these two risk genes. First, we explored the expression of transcription factors (TFs) disturbed by the two genes in the developmental brain and observed that the TFs are grouped into clusters involved in distinct cell types (Figure 6D). One cluster of TFs shows higher expression in neurons (c1), while other clusters of TFs are implicated in non‐neuronal cells, e.g., microglia (c2), astrocytes (c3, c5, c7). We then applied SCENIC^[^ ^48^ ^]^ to the three fetal single cell datasets^[^ ^45^, ^46^, ^49^ ^]^ to identify regulons that are active in neurons of developmental brain (Methods). For *CUL7*, four regulons, *CUX1, ONECUT2, PBX1, SREBF2*, were found to be active in neuronal cells (Table S11, Supporting Information); for *DCC*, identified regulons included *NEUROD1* (Figure 6E). Other regulons implicated in neuronal development and psychiatric disorders are listed in Table S12 (Supporting Information). These TFs and regulons have been repeatedly implicated in dendritic morphology and SCZ, supporting the mechanistic roles of *CUL7* and *DCC* in regulating these transcriptional programs in SCZ risk (see Discussion).

### 2.10 Further Validation Using PGC3

Finally, we utilized PGC3 as a validation resource to further examine our discoveries in this work. We inferred risk genes for GWAS of PGC3 and observed that dendrite‐related pathways turn out to be significant (PDD = 4.66 × 10^−3^, PDM = 1.69 × 10^−3^), aligning with our expectation that the dendrite‐related pathways are likely to be identified with the increasing sample size of GWAS. Among the 14 DD genes identified by CLOZUK+PGC, 4 of 4 sigGWAS genes remained significant in PGC3, and 5 of 10 subGWAS genes turned significant in PGC3 (Table S13, Supporting Information). For *DCC* and *CUL7* selected for iPSC validation, both are identified as the HRGs of the corresponding sigGWAS loci in PGC3.

## 3 Discussion

Although GWAS have successfully identified more than 200 loci associated with SCZ, the identified loci explain only a small proportion of disease heritability. Our understanding of SCZ genetic mechanisms is, however, still limited. In this study, we explored the strategy of empowering the discovery by including ‘sub‐threshold’ significant loci and identified DDM as a novel category of SCZ pathophysiology. Of note, the risk genes in the novel category have relatively weaker effects and are only detected in these subGWAS loci. Nevertheless, from multiple genetic angles, we showed that these sub‐threshold risk genes are significantly enriched in SCZ heritability and are robustly associated with the heritability of other neuropsychiatric disorders, including MDD and BP. We selected two sub‐threshold candidates, *DCC* and *CUL7*, to further investigate their cellular and molecular functions in hiPSC‐derived neurons and observed significant dendritic morphological abnormalities in neurons with perturbed expression of the candidate risk genes. Transcriptomic analysis of the perturbed neurons showed that disrupted functions are implicated in SCZ susceptibility, and identified key regulators, including *CUX1/2* and *NEUROG1*, involved in the disrupted regulatory networks.

*DCC* (Deleted in Colorectal Cancer) is a receptor protein critical for axonal development and guidance in the brain. It plays a key role in the formation and maturation of neural circuits, particularly in the prefrontal cortex (PFC) – a region essential for executive functions and emotional regulation.^[^ ^50^ ^]^ Extensive genetic and functional studies have established DCC's critical role in SCZ pathogenesis, primarily through its regulation of mesocorticolimbic dopamine circuitry. Specific risk‐associated SNPs (rs2270954 in the 3'UTR and rs2229080 in exon 3) contribute to elevated DCC expression by compromising mRNA stability and disrupting loss‐of‐heterozygosity‐mediated downregulation.^[^ ^51^, ^52^ ^]^ This DCC overexpression drives hyperdopaminergic states – a hallmark of SCZ – while its reduction confers neuroprotection, as demonstrated by both the resistance of DCC‐deficient mice to SCZ‐like behaviors^[^ ^53^, ^54^ ^]^ and the 50% reduction in DCC expression observed following haloperidol treatment in the ventral tegmental area.^[^ ^55^ ^]^ Our study provides direct evidence that DCC upregulation impairs neurite outgrowth in an iPSC‐derived neurons, offering mechanistic insight into its contribution to SCZ‐related circuitry dysfunction. The validation of CUL7 and DCC supports the implication of dendrite morphogenesis in SCZ, and genes in the related pathways warrant further mechanistic investigations and therapeutics development. For example, potential intervention strategies include DCC suppression or modulation of the netrin‐1 pathway, representing novel approaches in precision psychiatry.^[^ ^50^ ^]^

*CUL7*, encoding cullin‐7, plays a critical role in dendrite elaboration in neurons. It functions as part of a ubiquitin ligase complex, specifically CUL7‐RING (FBXW8), which is essential for the proper formation and development of dendrites, the branching extensions of neurons that receive signals.^[^ ^56^ ^]^ While the potential association between *CUL7* and SCZ remains largely unexplored, our findings suggest that its upregulation contributes to neurite loss and morphogenesis abnormality. These observations could indicate a possible role for *CUL7* in SCZ‐related neuronal connectivity alterations, though further validation is needed to elucidate its role in SCZ pathogenesis.

The networks dysregulated by *DCC* and *CUL7* involve key TFs implicated in dendrite morphogenesis and synapse function. *CUX1/2*, as an example, has been demonstrated to regulate dendritic branching, spine morphology, and synapse formation in upper cortical layers.^[^ ^57^ ^]^ *CUX1* is a cortical layer III marker and shows reduced expression in dendritic spine density in SCZ in iPSC‐derived cortical neurons derived from SCZ patients.^[^ ^58^ ^]^ *CUX1* also shows association with other neurodevelopmental disorders like developmental delay or intellectual disability.^[^ ^59^ ^]^ *NEUROD1* is a member of Neurod family, standing as a key regulator of neuronal progenitor cell differentiation and neuronal specification cortex.^[^ ^60^ ^]^ Upregulation of Neurod1 expression in forebrain improves learning abilities.^[^ ^61^ ^]^ If both Neurod1 and Neurod6 are mutated, the mice display several phenotypes, including small dendritic arborization and alterations of the entorhinal and commissural axonal projections.^[^ ^62^ ^]^ The multiple lines of evidence supporting the involvement of TFs in SCZ suggest that SCZ risk alleles’ effects on *DCC* and *CUL7* likely disrupt these key regulatory networks that ultimately lead to abnormal dendritic morphogenesis.

In this study, we utilized a subGWAS‐based approach to identify and prioritize candidate genes, including CUL7 and DCC, for their involvement in dendrite development in SCZ. While this strategy enabled the identification of suggestive associations under relaxed significance thresholds, we recognize its inherent limitations. The reduced statistical stringency increases the risk of false positives, necessitating cautious interpretation of results. Additionally, many identified variants reside in noncoding regions with limited functional annotations, posing challenges for mechanistic interpretation. Although the iRIGS framework integrates genomic, genetic, and network‐based evidence to prioritize risk genes, the biological mechanisms remain incompletely resolved. These findings should be regarded as preliminary until replicated in larger cohorts or experimentally validated in model systems. While our expression profiling analyses of CUL7 and DCC overexpression in iPSC‐derived neurons support their potential roles in dendrite development—a process implicated in SCZ—such correlative evidence is insufficient to establish direct causality. Additional experiments are warranted to dissect the causal role of the genes in SCZ risk. Advanced CRISPR‐based technologies, such as CRISPRa/i,^[^ ^63^ ^]^ can be employed to enhance/inhibit the expression of the target genes. Perturb‐Seq, involving CRISPRa/i technologies, has unique advantages to assess the gene perturbation effects of hundreds or thousands of genes, in parallel.^[^ ^64^ ^]^ New advances in enhancer CRISPR technologies^[^ ^65^, ^66^ ^]^ can manipulate the activity of enhancers containing the SCZ‐associated variants to directly assess the effects of the variants and their target risk genes. Complementary causal inference methods, such as Mendelian randomization and colocalization analyses, would additionally strengthen mechanistic insights. In vivo experiments in animal models can further establish the causal roles of the risk variants and genes in SCZ pathogenesis. Altogether, these strategies will help bridge the gap between genetic associations and biological mechanisms in SCZ.

Dendrite development and morphogenesis are critical determinants of synaptic functions. Dendrites are the major sites for synaptic connections,^[^ ^67^ ^]^ and dendritic spines specialized from the shaft, contain postsynaptic receptors for the vast majority of excitatory glutamatergic synapses.^[^ ^68^ ^]^ Conversely, stabilization of dendrites also requires productive synaptogenesis and active synaptic input.^[^ ^68^, ^69^, ^70^ ^]^ Therefore, dendrites and synapses contribute to the development and function of each other. Synaptic dysfunction is long implicated in SCZ.^[^ ^7^, ^23^, ^24^, ^71^, ^72^, ^73^, ^74^ ^]^ Furthermore, synaptic loss and dendritic atrophy have been reported in SCZ, as well as in other neurodevelopmental and neurodegenerative disorders such as MDD and Alzheimer's disease (AD),^[^ ^75^ ^]^ suggesting that maintenance of normal dendritic architecture and restoration of related functions, such as synapse development, may represent viable therapeutic strategies. For instance, treatment regimens that prevent synaptic over‐pruning during childhood and adolescence could benefit patients with neurodevelopmental disorders.^[^ ^76^ ^]^ Indeed, a previous study has shown that patients chronically exposed to minocycline, an antibiotic with anti‐inflammatory effects that reduces pruning, are at significantly lower risk of incident psychosis.^[^ ^77^ ^]^ Moreover, restoration of DDM in adulthood may pave a path to successful treatment. We note that second‐generation antipsychotic drugs such as olanzapine and clozapine can enhance neurite outgrowth and increase spine numbers.^[^ ^78^, ^79^ ^]^ In addition, another study demonstrated an exciting strategy of designing a synthetic synaptic organizer protein to restore dendritic spine numbers, which finally led to the improvement of hippocampus‐dependent learning in AD.^[^ ^80^ ^]^ Moreover, they anticipated that other extracellular scaffold proteins could be used to restore synaptic connectivity in other neurodevelopment and neurodegenerative diseases. Given the fact that therapeutics with direct genetic evidence are more likely to be successful,^[^ ^81^, ^82^ ^]^ such therapies to restore and stabilize dendrite morphogenesis are an important area for further exploration.

Although dendritic alterations have long been observed in SCZ and other psychiatric conditions,^[^ ^83^ ^]^ little is known about the link between genetic risk and dendritic dysfunction. In this study, we discovered, validated, and experimentally confirmed in hiPSC models dendrite dysmorphogenesis in SCZ. Our results are consistent with the conclusion that SCZ‐associated genetic variants are significantly correlated with reduced neurite density index (NDI) across cortical, subcortical, and white matter regions, suggesting impaired myelinated axon and dendritic arborization as a potential neurodevelopmental mechanism in SCZ pathogenesis.^[^ ^84^ ^]^ This reflects the effect size heterogeneity of different genetic mechanisms in SCZ risk, with dendrite morphogenesis being a relatively weaker mechanism, in contrast to the major neuronal functions strongly implicated in sigGWAS loci. The heterogeneity between loci with different effect sizes leads to the discovery of mutually exclusive functional modules, even though dendrite development and synapse development are molecularly correlated. Most human complex diseases are highly heterogeneous, and identified GWAS loci are far from complete due to the relatively limited sample sizes in most studies. Therefore, many gene modules with weaker effect sizes remain to be discovered. Our present strategy demonstrates that focusing on subGWAS signals constitutes a powerful and valuable approach to identify categories of functional gene sets.

In this study, we prioritized CLOZUK+PGC for discovery and PGC3 for validation to underscore the potential of subGWAS as an alternative approach when GWAS sample sizes are limited. Notably, DCC and CUL7—validated experimentally—were later confirmed as HRGs in PGC3, reinforcing our findings. While PGC3 could serve as a discovery cohort, our design highlights how subGWAS can uncover biologically relevant signals even in smaller datasets. Future work will explore PGC3's subGWAS loci to identify pathways with weaker effects.

The selection of an appropriate subGWAS threshold is an important point in our framework. We have systematically evaluated subGWAS thresholds of 1 × 10^−5^, 5 × 10^−6^, and 1 × 10^−6^ to balance sensitivity and specificity in identifying risk loci. The number of candidate loci under these thresholds is 457, 343, and 180, respectively. While our final choice of 1 × 10^−6^ is more stringent than thresholds used in comparable studies,^[^ ^15^, ^16^, ^85^ ^]^ it ensures a manageable number of high‐priority loci for downstream analysis while improving reliability. Notably, dendrite pathway genes—central to our findings—were consistently identified as high‐risk genes (HRGs) even at the 5 × 10^−6^ threshold, underscoring the robustness of our major results. We acknowledge that relaxing the threshold would increase the absolute number of false positives, introducing additional noise into the analysis. Importantly, the fraction of true positives is likely higher under stringent thresholds, as looser thresholds disproportionately amplify noise. Future refinements could integrate multi‐dimensional evidence—such as allele effect consistency across studies, single‐cell epigenomic profiles, high‐resolution chromatin interactions, and functional annotations—to further enhance the reliability of subGWAS loci selection.

## 4 Experimental Section

### Replication of PGC subGWAS Variants From the CLOZUK+PGC and PGC3

The 52 new sigGWAS loci were extracted from the CLOZUK+PGC study.^[^ ^8^ ^]^ For each index single nucleotide polymorphism (SNP) of the 52 loci, it was extended to a linkage disequilibrium (LD) region by including other SNPs tagged by the index SNP with an *r* ^2^ threshold defined by HaploReg V4.^[^ ^86^ ^]^ When requiring *r* ^2^ ≥ 0.9, 38 index SNPs from the 52 loci were extended to LD regions (with the rest having no available SNPs in LD). With a *loose* criterion of *r* ^2^ ≥ 0.2, all 52 index SNPs were extended to valid LD regions. An analysis was performed to determine how many of the 52 loci had subGWAS signals in PGC study. The same procedures were replicated for the new sigGWAS in PGC3.

### Defining Independent subGWAS Index SNPs for CLOZUK+PGC Data as Input to iRIGS

The GWAS summary statistics were collected from the CLOZUK+PGC study, and an iterative strategy was utilized to obtain independent subGWAS index SNPs:

In step 1, all the SNPs were removed within ± 500 kb intervals centered on sigGWAS index SNPs to avoid potential overlaps.

In step 2, the SNP with the lowest *P* value was selected from the remaining SNPs, and then all the other SNPs within its ± 500 kb region were removed.

In step 3, step 2 was iterated until no further SNPs were left.

In step 4, the selected SNPs with 5 × 10^−8^ < *P* ≤ 1 × 10^−6^ were retained, and these SNPs were defined as subGWAS index SNPs.

### In Total, 180 subGWAS Index SNPs from the CLOZUK+PGC Data Enhancers in GWAS LD Regions

For each index SNP, either from sigGWAS or subGWAS, a threshold of *r* ^2^ was adopted to define a LD region. Then DLPFC enhancer data were collected from the ROADMAP Epigenomics Project.^[^ ^87^ ^]^ To evaluate whether sigGWAS or subGWAS LD regions significantly overlap with DLPFC enhancers, the strategy proposed in a previous study was followed to construct a set of background SNPs used for a permutation test.^[^ ^15^ ^]^ Specifically, for either sigGWAS or subGWAS, a matched background SNP list was picked requiring that it has the same number of SNPs as sigGWAS/subGWAS and further each SNP in the background SNP list has similar features to a given index SNP in sigGWAS/subGWAS: matched minor allele frequency (±0.1), gene density in 1 Mb region centered at the selected SNP (±3), location of the picked SNP (whether located in a gene body), distance to TSS of the closest protein‐coding gene (±25 kb) and if the LD region of the selected SNP contains a similar number of related SNPs under the same criterion, i.e., *r* ^2^ ≥ 0.2 (±5). Randomly, 1000 matched background SNP lists were picked.

Three statistics are presented in the related section above:

First, the number of LD regions overlapping ≥ 1 DFPLC enhancers in sigGWAS/subGWAS and 1000 random background SNP lists was counted, respectively. *P*‐values were calculated using the observed numbers in sigGWAS/subGWAS compared to the background of random lists.

Second, the mean number of enhancers overlapping each LD region in sigGWAS/subGWAS and 1000 random lists was also calculated. *P*‐values were calculated based on the observed mean and background means.

Third, enhancers from other tissues in addition to DFPLC were collected from the ROADMAP Epigenomics Project. To obtain clean results on tissues other than brain, brain enhancers were removed from all non‐brain tissues. Then for a given tissue, the z score for the *i*th SNP in sigGWAS/subGWAS was calculated using the formula:

(1)

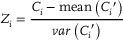

where *C_i_* is the number of enhancers overlapped by the *i*th SNP LD region in sigGWAS/subGWAS, *C_i_*’ is the number of enhancers overlapped by the corresponding LD regions for matched SNPs sampled. Z score > 0 means a given test SNP overlaps more enhancers than its random background samples, and vice versa.

### Applying iRIGS to Identify HRGs for SCZ

HRGs for SCZ were identified by applying iRIGS,^[^ ^10^ ^]^ a Bayesian framework that integrates multiple features and a gene network to predict risk genes affected by GWAS loci. The same gene network and features from their study were adopted in the analyses conducted here. Integrated features include the distance from target gene to index SNP (DTS), DNMs identified in SCZ (Turner *et al.*, 2017, differential expression between SCZ patients and controls (Fromer et al., 2016), and distal regulatory elements (DREs)‐promoter links (Andersson et al., 2014; Won et al., 2016; Mifsud et al., 2015). Please refer to the iRIGS study for details on how to generate these features and gene network.

### LDSC Analysis

SNPs in the defined region (±10 kb/±100 kb) centered around gene TSS were extracted, and the LDSC software was downloaded from https://github.com/bulik/ldsc. Note that DTS (distance of SNP to gene TSS) used in iRIGS was a confounding factor for LDSC, since genes closer to index SNPs were more likely to have a high LDSC score. To avoid this confounding effect, DTS was excluded in the application of iRIGS to predict HRGs for LDSC enrichment analysis.

### Gene Set Enrichment Analysis

Gene sets previously implicated in SCZ were collected as in.^[^ ^10^ ^]^ The gene sets include: FMRP targets; postsynaptic density (PSD) proteins; genes related to presynaptic proteins (PRP), PRAZ, and synaptic vesicles (SYV); the GABA_A_ receptor complex; CCS genes; targets of miR‐137; genes from the database AutDB; evolutionarily constrained genes (ECG); essential genes; ASD genes implicated by transmission and *de novo* association (TADA) test analysis; activity‐regulated cytoskeleton‐associated proteins (ARC), mGluR5 genes, and NMDAR; and targets of RNA binding protein, fox‐1 homolog 1 (RBFOX1), a brain‐ and muscle‐specific splicing factor. *P*‐values were computed by a hypergeometric test and corrected using the Bonferroni method.

Terms from the MPO^[^ ^88^ ^]^ were collected from MGI (http://www.informatics.jax.org/). In that study, only central nervous system terms were analyzed, and the method here was modified by including all terms to get a more comprehensive phenotypic profile. Briefly, gene list of a specific term was determined by including all the genes in itself and its descendant terms. In this study, 12 490 terms were compiled in total, and 1721 terms with >50 genes were selected for the analysis. *P*‐values were computed by hypergeometric tests and corrected for multiple testing using the Bonferroni method.

GO terms were downloaded from Gene Ontology database,^[^ ^89^ ^]^ and terms were grouped following the relationships of “is_a,” “part_of”, and “regulates,” i.e., one term is a descendant term of a parent term if it has any of the three kinds of relationships with its parent term. In the hierarchical structure, genes of one term include all the genes residing in its descendant terms. *P* values were calculated using clusterProfiler^[^ ^90^ ^]^ and the false discovery rate (FDR) method was used for multiple testing correction.

### Gene Expression Analysis

The transcript per million (TPM) values of genes were downloaded from the GTEx portal (https://www.gtexportal.org/), and tissue specificity was measured by Preferential Expression Measure (PEM).^[^ ^91^, ^92^ ^]^ Accounting for the fact that tissues from the same organ may be correlated with each other and underlie the same disease, the tissue specificity in a grouping manner was calculated. For a specific tissue, its PEM was computed versus all tissues in other organs; for example, the specificity of the frontal cortex was calculated by comparing it with all other non‐brain tissues.

To calculate developmental‐stage specificity, the data from BrainSpan (www.brainspan.org) were downloaded. Similarly, the stages were grouped into two major ones: prenatal (S2–S7) and postnatal stages (S8‐S14), and PEM was calculated for each stage following the same grouping manner, i.e., each stage in a prenatal category was computed comparing only with postnatal stages, and vice versa.

To calculate the specificity of brain cell types, four sets of single‐cell sequencing (scRNA‐seq) data were collected from previous studies, including two of fetal brain^[^ ^45^, ^46^ ^]^ and two of adult brain.^[^ ^45^, ^93^ ^]^ Cell types were annotated in the original studies, including excitatory neuron, inhibitory neuron, astrocyte, microglia, and others. The specificity of each gene in neurons was calculated following the same strategy used in a previous study,^[^ ^94^ ^]^ i.e., the mean expression in neuronal cells divided by the sum of mean expression in all cell types. Note that in these studies, sub‐clusters (e.g., excitatory neurons in prefrontal cortex and inhibitory neurons in hippocampus) were annotated for excitatory/inhibitory neurons, so first the specificity for these sub‐clusters was calculated and then grouped them into two large categories, i.e., excitatory and inhibitory neurons.

### Determine Direction of Expression the Risk Alleles of HRGs

Because direct eQTLs among the majority of subGWAS index SNPs and HRGs were missing, the risk direction of expression of HRGs was identified using all the SNPs in the LD region. First, all the LD_SNPs of a subGWAS SNP were extracted using the criterion of LD > 0.1. Second, the eQTLs involving the LD_SNPs and HRGs were identified. After matching the risk allele of LD_SNPs, the directions of how risk alleles affect the expression of HRGs were obtained. Because there were many LD_SNPs mapping to a single subGWAS SNP, the directions of all the LD_SNPs were summarized, and the major direction was taken as the risk direction of HRGs.

### Differentiation of iPSCs into Forebrain‐Specific Neural Progenitors and Cortical Neurons

Cortical neuronal differentiation from a healthy control human hiPSC line (WTC11; Coriell GM25256) was adapted from a previously established protocol.^[^ ^95^, ^96^, ^97^ ^]^ Briefly, hiPSCs colonies were detached with 1 mg/mL collagenase (Thermo Fisher Scientific) treatment for 30 min and suspended in embryonic body (EB) medium, consisting of bFGF‐free hiPSC medium supplemented with 2 µM Dorsomorphin (Tocris) and 2 µM A‐83 (Tocris), in non‐treated polystyrene plates for 4 days with a daily medium change. After 4 days, EB medium was replaced by neural induction medium (NPC medium) consisting of DMEM/F12 (Thermo Fisher Scientific), 1X N2 supplement (Thermo Fisher Scientific), 1X MEM NEAA (Thermo Fisher Scientific), 2 µg/mL heparin (Sigma) and 2 µM cyclopamine (Tocris). The floating EBs were then transferred to Matrigel (Corning)‐coated 6‐well plates at day 7 to form neural tube‐like rosettes. The attached rosettes were kept for 15 days with NPC medium change every other day. On day 22, the rosettes were picked mechanically and transferred to low attachment plates (Corning) to form neurospheres in NPC medium containing 1X B27 (Thermo Fisher Scientific). The neurospheres were then dissociated with Accutase (Thermo Fisher Scientific) and placed onto Poly‐D‐Lysine/laminin (Sigma)‐coated coverslips in the neuronal differentiation medium, consisting of Neurobasal medium (Thermo Fisher Scientific) supplemented with 1X Glutamax (Thermo Fisher Scientific), 1X B27 (Thermo Fisher Scientific), 1 µM cAMP (Sigma), 200 ng/mL L‐Ascorbic Acid (Sigma), 10 ng/mL BDNF (PeproTech), and 10 ng/mL GDNF (PeproTech).

### Genetic Manipulations and Morphological Analyses of hiPSC‐Derived Cortical Neurons

Lentiviruses for overexpression of *CUL7* (pLV‐EF1A>hCUL7[NM_001374872.1]), DCC (pLV‐EF1A>hDCC[NM_005215.4]), or control (pLV‐EGFP:T2A:Puro‐EF1A>mCherry) were purchased from VectorBuilder (Chicago, IL). Human hiPSC‐derived cortical neurons were infected with lentiviruses (MOI = 2) at day 4 after differentiation. At day 10, neurons were fixed with 4% paraformaldehyde (Sigma) for 15 min at room temperature. Samples were permeabilized and blocked with 0.25% Triton X‐100 (Sigma) and 10% donkey serum in PBS for 20 min as previously described.^[^ ^95^ ^]^ Samples were then incubated with an anti‐MAP2 primary antibody (Rabbit; 1:1000; Millipore AB5622) at 4 °C overnight, followed by incubation with secondary antibody (anti‐Rabbit Alexa Fluor 568; 1:1000; ThermoFisher A10037) for 1 h at room temperature. Images were taken by a Nikon Eclipse Ti‐E microscope and acquired from three or four independent cultures with identical settings for parallel cultures. The neurite lengths of individual neurons were measured using ImageJ with NeuronJ plugin (NIH) according to developer's instructions.

### RNAseq Experiment and Analysis

Neurons were homogenized in TRIzol Reagent (Invitrogen, 15596018), and RNA was isolated using the Direct‐zol RNA Miniprep Kit (Zymo, R2052) according to the manufacturer's instructions. An Illumina mRNA sample prep kit (cat. no. RS‐100‐0801) was used to construct the RNA‐seq library, following the manufacturer's instructions. Poly‐T oligo‐attached magnetic beads were used to purify poly‐A‐containing mRNA, which was then fragmented into small pieces using divalent cations at elevated temperatures. The cleaved RNA fragment was copied into the first strand of cDNA using reverse transcriptase and random primers. Subsequently, DNA Polymerase I and RNase H were used to synthesize the second‐strand cDNA. The resulting cDNA fragments underwent a terminal repair process, during which a single “A” base was added and an adapter was ligated. The gel‐purified products were used to create the final cDNA libraries, which were then enriched by PCR. The size and concentration of the library constructs were verified by running them on a bioanalyzer before sequencing them on an Illumina HiSeq6000. The quality of the raw reads was analyzed to ensure that library generation and sequencing were suitable for further analysis. Reads obtained are trimmed using Trimmomatic^[^ ^98^ ^]^ to remove adapters. Then they are aligned to hg38 genome using HISAT2^[^ ^99^ ^]^ and summarized to read count using featureCounts.^[^ ^100^ ^]^ Differential expression analysis was performed using DESeq2^[^ ^101^ ^]^ with FDR < 0.05 and lfcThreshold setting to log_2_(1.1). To ensure sufficient statistical power for downstream regulon analysis (SENIC), a relaxed fold change threshold (FC > 1.1) was adopted while maintaining FDR < 0.05. The majority of results remained robust when using the more stringent log2(1.2) cutoff, confirming the stability of the findings across threshold choices.

### Transcription Regulatory Network Analysis

The count matrix of the three fetal single cell datasets for each DE list was extracted, and then SCENIC was applied to the count matrices. To determine the neuron‐preferred regulons, AUCscore, which was the measurement of a regulon's activity in each cell, was first used to generate a binarization matrix based on a threshold automatically derived from the distribution of the AUCscore of each cell type. Second, the percentage of cells in each cell type where a regulon was active was determined using the binarization matrix. Third, the neuron‐preferred regulons were selected, but subtypes of neurons did not matter in this case. To achieve this goal, a regulon's specificity was calculated using a group strategy. All cells were divided into two groups, i.e., neuron‐related and non‐neuron‐related groups. The specificity score for a particular neuron‐related cell type was calculated using only that specified neuron‐related cell type and all the non‐neuron‐related cell types, ignoring all the other neuron‐related cell types in the same group. Vice versa, the same approach was applied for the non‐neuron‐related group. Supposing there were i neuron cell types and j non‐neuron cell types in total, to calculate the specificity score of the kth cell type

(2) $$\mathrm{Specificity}\_\mathrm{Scor}e_{k}=\left\{\begin{array}{c} \frac{P_{k}}{\mathrm{sum}\left(P_{j}\right)+P_{k}},if\;k\in \left(1,2,\cdot \cdot \cdot ,i\right)\\ \frac{P_{k}}{\mathrm{sum}\left(P_{i}\right)+P_{k}},if\;k\in \left(1,2,\cdot \cdot \cdot ,j\right)\; \end{array}\right.$$

*P_i_*,*P_j_*,*P_k_* are the percentages of active cells in each cell type for a regulon.

Fourth, a neuron‐preferred regulon is defined if its specificity score in neuron‐related cell types ranks in top 2 across all cell types in each study.

### Statistical Analysis

Data pre‐processing did not involve transformation or normalization unless specified, and outliers were evaluated but retained. Results are presented as mean ± SD unless otherwise noted. The default test was a one‐sided Wilcoxon rank‐sum test unless otherwise indicated. For subGWAS loci‐enhancer overlaps, random sampling tests, for heritability enrichment were applied, LDSC regression was used, and for spatiotemporal expression patterns, one‐sided Wilcoxon tests were used. Gene set enrichment analysis (GSEA) with 18 prior‐knowledge gene sets used two‐sided Fisher's exact tests with Bonferroni correction (α = 0.05), while GSEA with MPO/GO terms employed Q‐value correction. Rare coding variants were assessed via random sampling tests, and dendrite length differences were analyzed using one‐sided t‐tests (n = 70 per condition). Sample sizes (N) for each analysis are detailed in the main text. Analyses were conducted in R with appropriate packages, and LDSC used the official software. Parametric tests assumed normality, and multiple testing corrections were applied as specified (α = 0.05 unless otherwise noted).

## Conflict of Interest

The authors declare no conflict of interest.

## Author Contributions

R.C., B.S., and Q.W. are co first‐author and contributed equally to this work. R.C., Z.W., and B.L. conceived the overall design of the study. R.C. and Q. Wang conducted most of the data analyses. B.S., C.X., and Z.W. performed the hiPSC related experiments and analyses. Q.W., Y.J., H.Y., X.Z., and F.C. provided data integration and analyses. J.S.S., E.H.C., and N.J.C. contributed to the interpretation of the results. R.C., Q.W., Z.W., and B.L. wrote the manuscript, and all authors participated in the review and revision of the manuscript.

## Supporting information



Supplemental FigureS1‐S24

Supplemental TableS1‐S13

## Data Availability

The data that support the findings of this study are openly available in Dropbox at https://www.dropbox.com/scl/fo/mj5tp87vpnle768snhrvz/AMt2k9Yfz7EuTZrj7zpl‐Tc?rlkey=a3f89jyeve69niaz18799l60k, reference number 0.
