# Supplementary material for: Analyses of GWAS and Sub‐Threshold Loci Lead to the Discovery of Dendrite Development and Morphology Dysfunction Underlying Schizophrenia Genetic Risk
Source: Adv Sci (Weinh). 2025 Aug 29;12(39):e08519. doi: 10.1002/advs.202508519 (PMC12533335; doi:10.1002/advs.202508519)
Supplement: Supplementary file 1 — Supplemental FigureS1‐S24 [file ADVS-12-e08519-s002.docx]

B

A


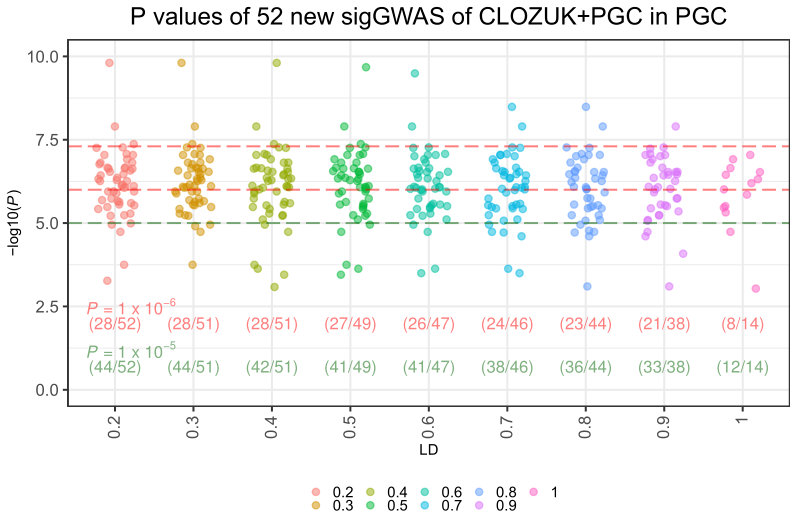


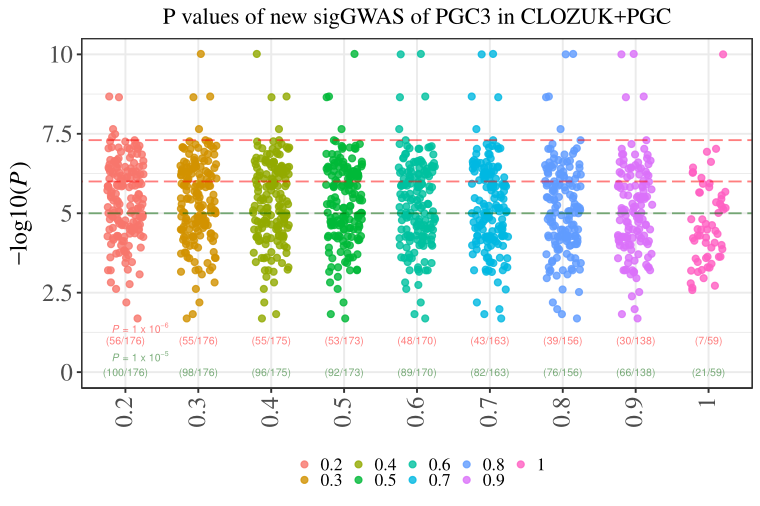


Figs. S1 The corresponding P values in PGC of the newly identified SigGWAS loci in CLOZUK+PGC (A) and PGC3 (B). The plot shows that a big portion of new sigGWAS loci of CLOZUK+PGC (A) and PGC3 (B) has at least one LD correlated variant with P values falling in different subGWAS hresholds (5 x 10^-8^ < P ≤ 1 x 10^-6^ or 5 x 10 ^-8^ < P ≤ 1 x 10^-5^) at different LD thresholds ([0.2, 1]) in their former studies.


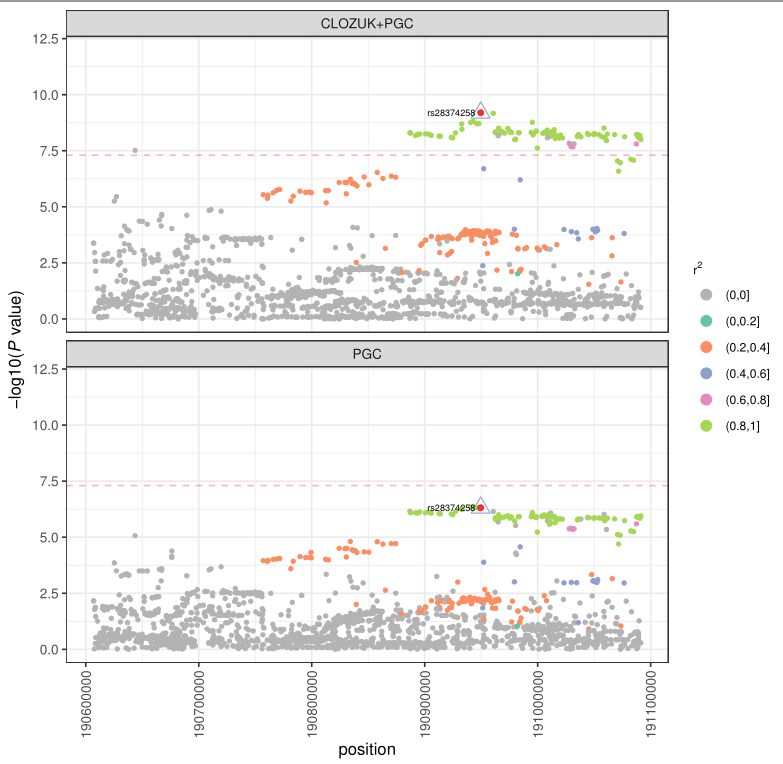


Figs. S2 An example of that subGWAS locus (rs28374258) in PGC become sigGWAS locus in CLOZUK+PGC. Different colors indicate the SNPs in different LD regions with the subGWAS locus.

A

B


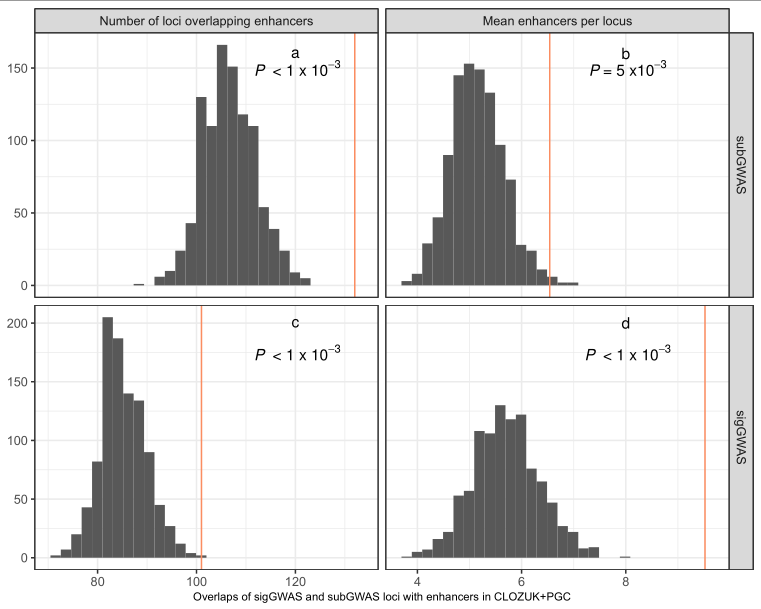

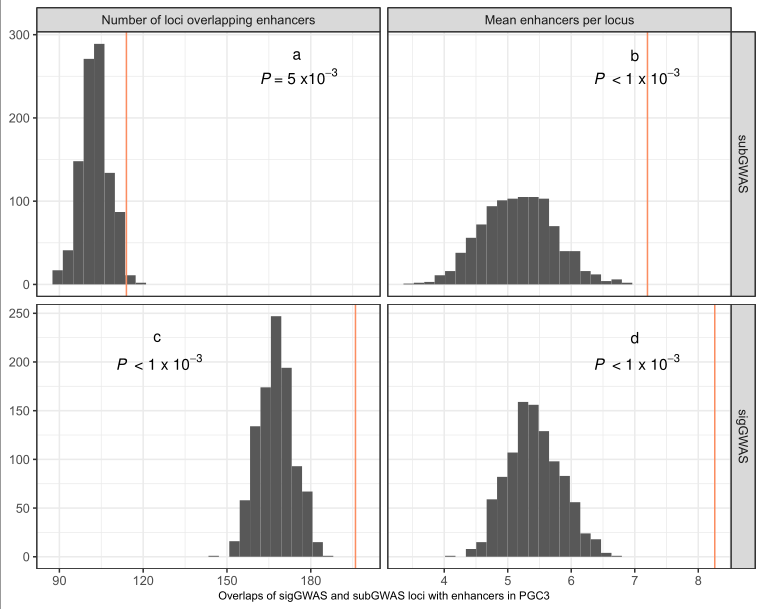


Figs. S3 Overlpas of sigGWAS and subGWAS loci with enhancers in ClOZUK+PGC (A) and PGC3 (B).

Panel A: (a, b) Number of loci in 145 sigGWAS (a, red vertical line) and 180 subGWAS (b) overlapped with enhancers of DLPFC comparing against 1000 permutation of randomly selected control loci lists. (c, d) Mean enhancers overlapped by sigGWAS (c) and subGWAS (d) comparing against to random controls.

Panel B: (a, b) Number of loci in 287 sigGWAS (a, red vertical line) and 188 subGWAS (b) overlapped with enhancers of DLPFC comparing against 1000 permutation of randomly selected control loci lists. (c, d) Mean enhancers overlapped by sigGWAS (c) and subGWAS (d) comparing against to random controls.

B

A


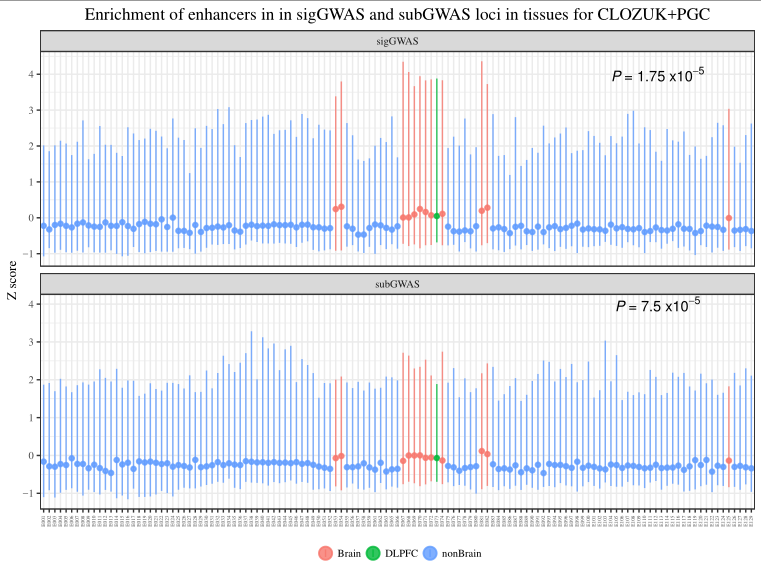


a

b


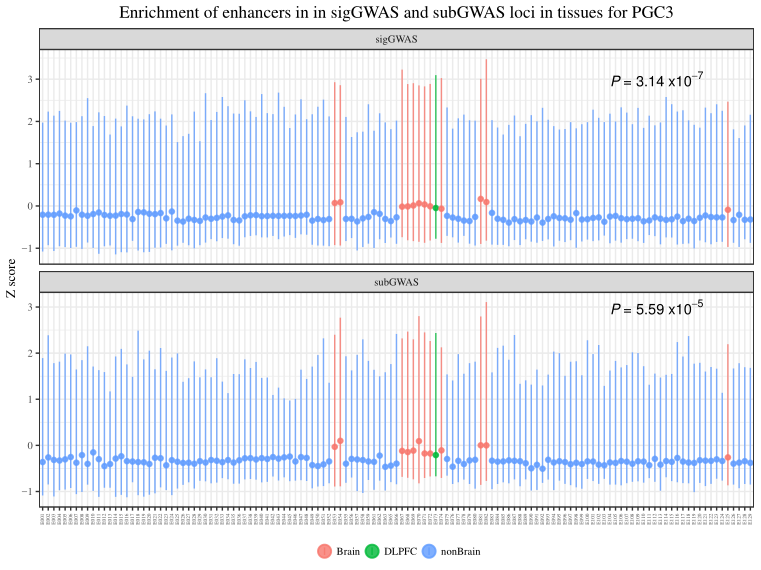


a

b

Figs. S4 Enrichment of enhancers in subGWAS loci regions in brain.

Panel A: results for CLOZUK+PGC. a. 145 sigGWAS. b. 180 subGWAS. Blue: nonbrain tissue; Red: Brain tissue; Green: DLPFC. The error bar plot shows the median and the 5th and 95th percentiles. Wilcoxon test, if not specified.

Panel B: results for PGC3. a. 287 sigGWAS. b. 188 subGWAS. Blue: nonbrain tissue; Red: Brain tissue; Green: DLPFC. The error bar plot shows the median and the 5th and 95th percentiles. Wilcoxon test, if not specified.


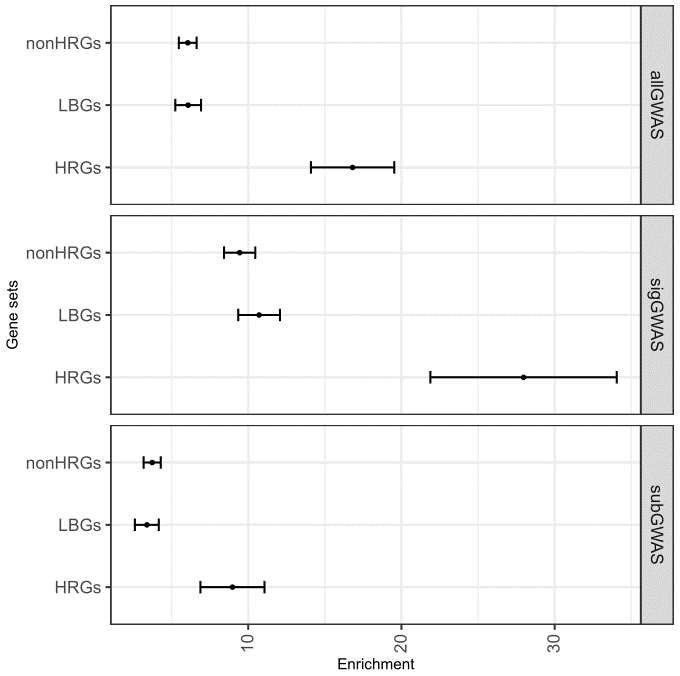


Figs. S5 LDSC analysis of HRGs excluding distance from the input features using regions ±10kb centered gene promoters. The center values represent the enrichment, and the error bars indicate standard errors.


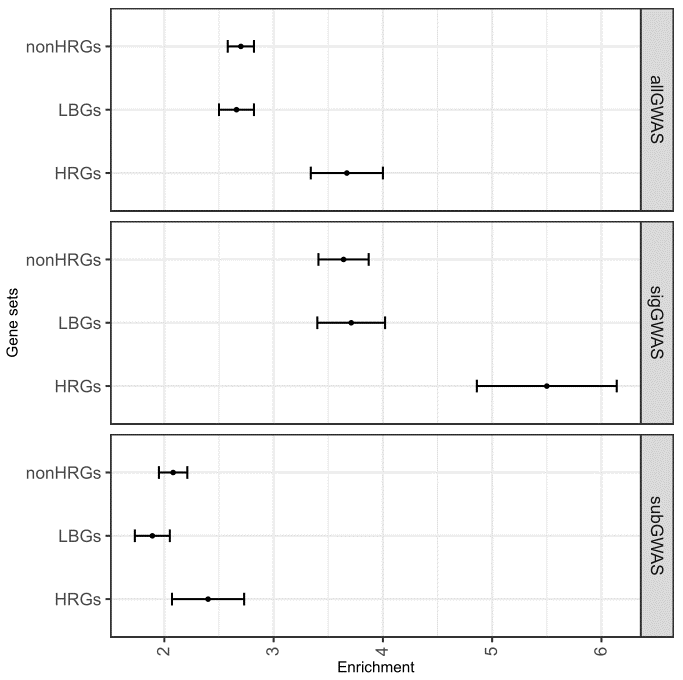


Figs. S6 LDSC analysis of HRGs excluding distance from the input features using regions ±100kb centered gene promoters. The center values represent the enrichment, and the error bars indicate standard errors.


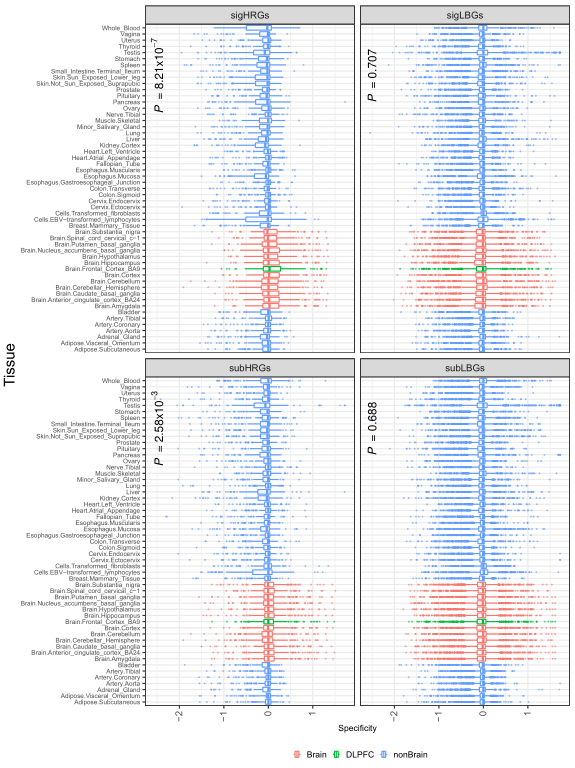


Figs. S7 GTEx_2015_adult brain specificity of sigHRGs and SubHRGs. The box plots show the median and the 25th and 75th percentiles. The whiskers extend from the box to the largest and smallest values no further than 1.5 times the inter quartile range (IQR) from the box (or the distance between the 25th and 75th percentiles). These are applied to all boxplot otherwise specified. Wilcoxon test, onesided.


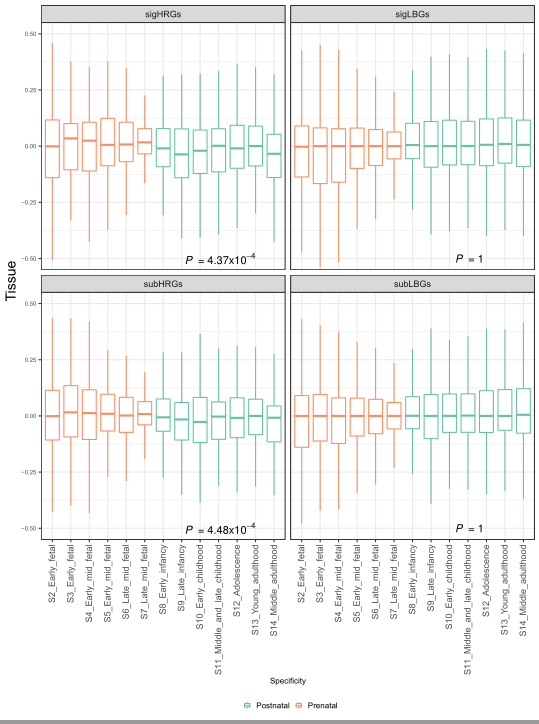


Figs. S8 SigGWAS and subGWAS shows early-stage expression in BrainSpan. Wilcoxon test, onesided.


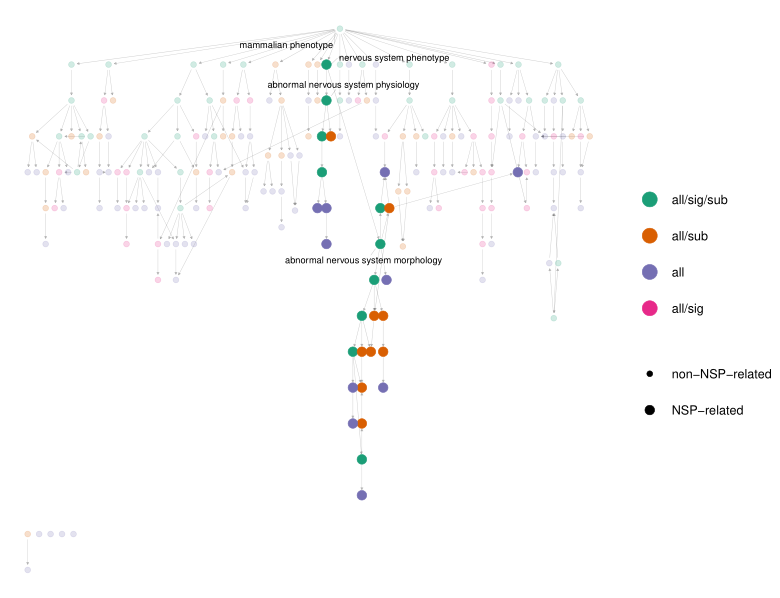


Figs. S9 DAG plot of enrichment of MPO. Longest branch in the plot is the term of **nervous system phenotype**.


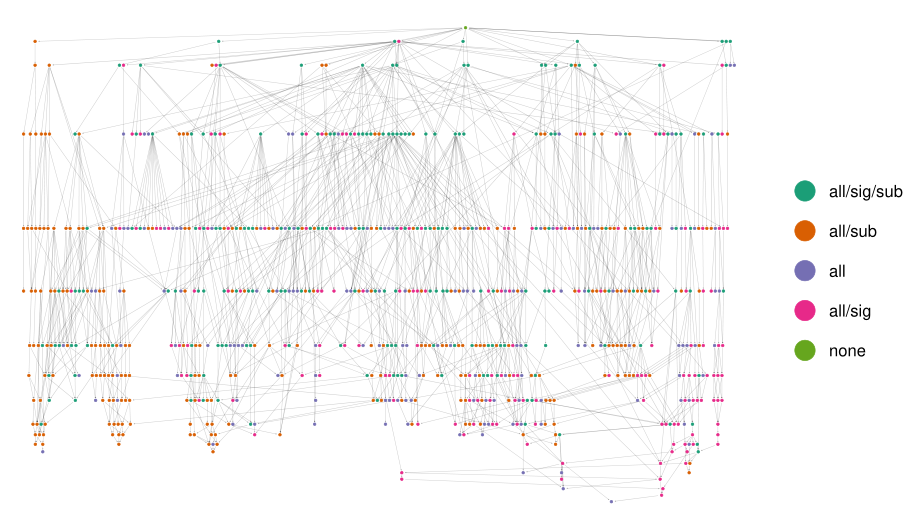


Figs. S10 All biological progress terms in GO analysis.


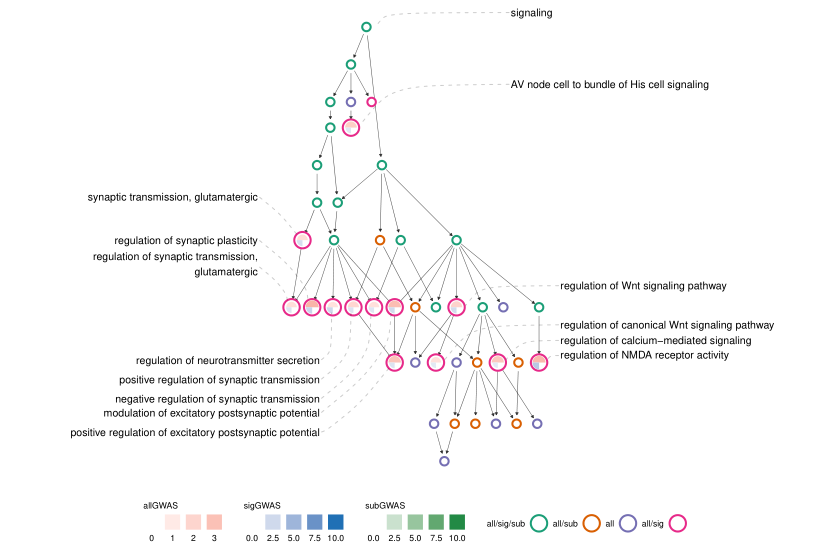


Figs. S11 Signaling term cluster. SigGWAS is likely to enrich in synaptic signaling terms. The -log_10_(P) of enrichment is represented by gradient colors, which is applied to other DAGs if not specified.


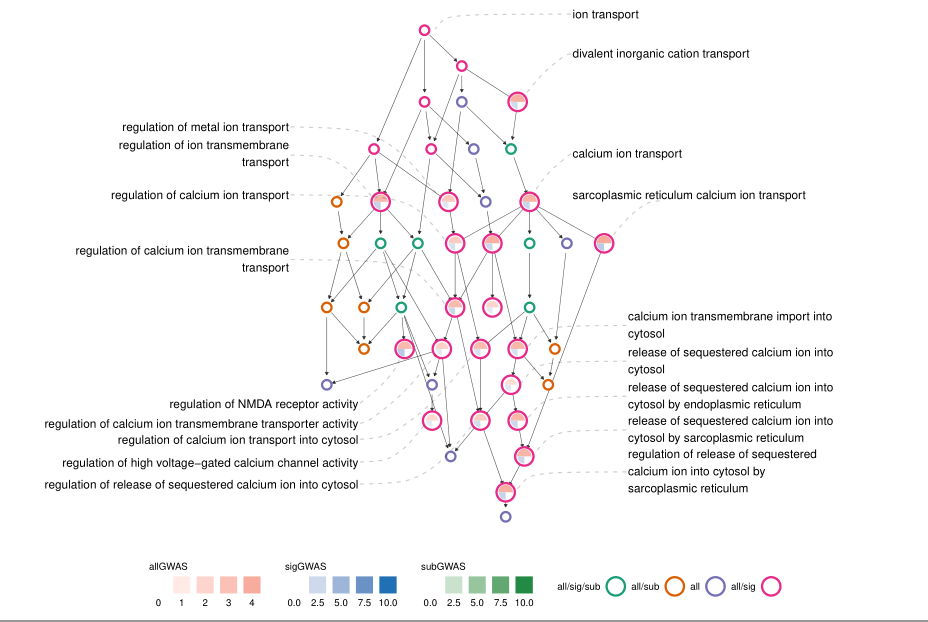


Figs. S12 ion transport terms. SigGWAS mainly enriched in calcium ion related terms.


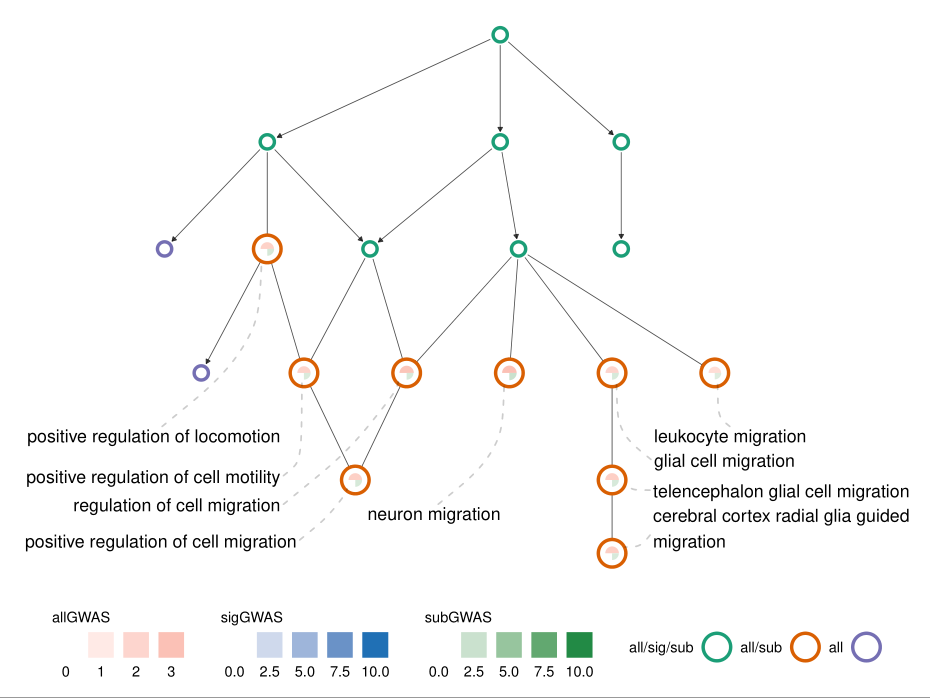


Figs. S13 locomotion terms


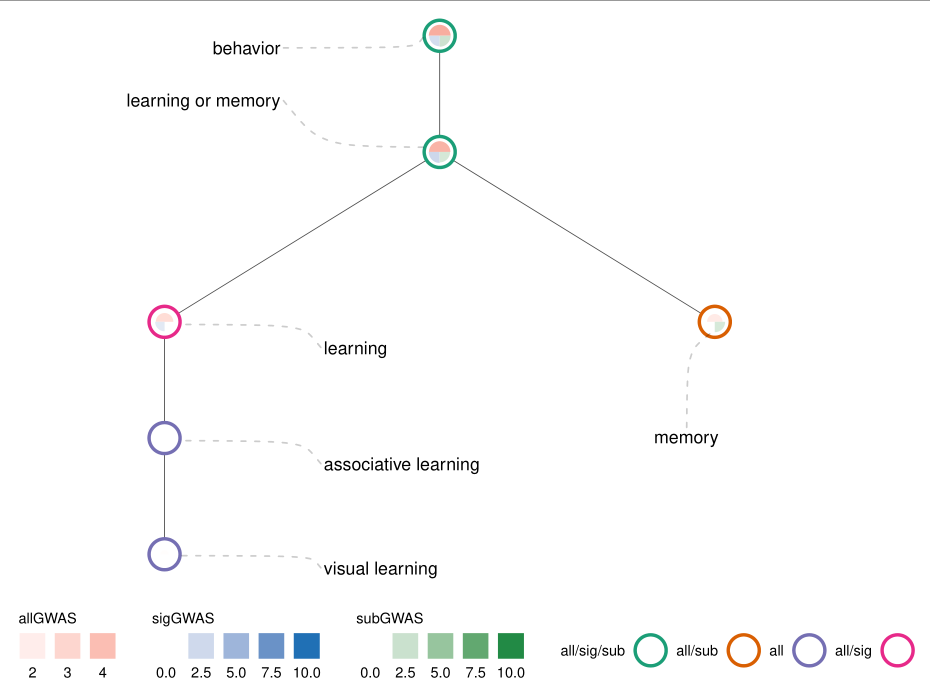


Figs. S14 Behavior terms


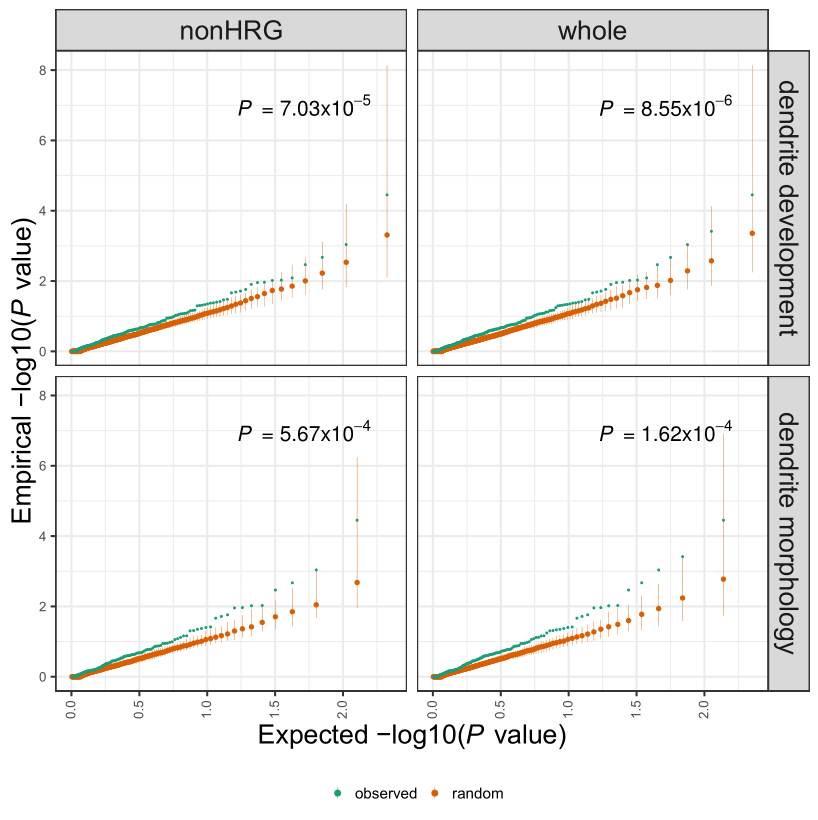


Figs. S15 QQ plot of -log10(P) of rare variants of DD and DM genes comparing to random sampled background (orange). The error bar plot shows the median and the 5th and 95th percentiles. Wilcoxon test; One-sided


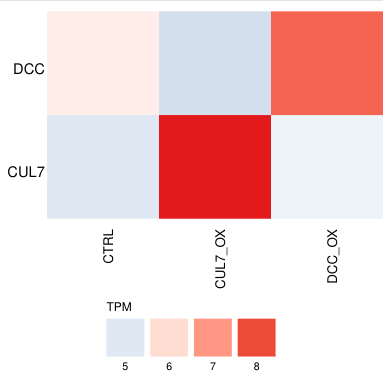


Figs. S16 The expressions of each targeted gene in each mutant. Comparing to the CTRL, the corresponding genes are up-regulated as expected.


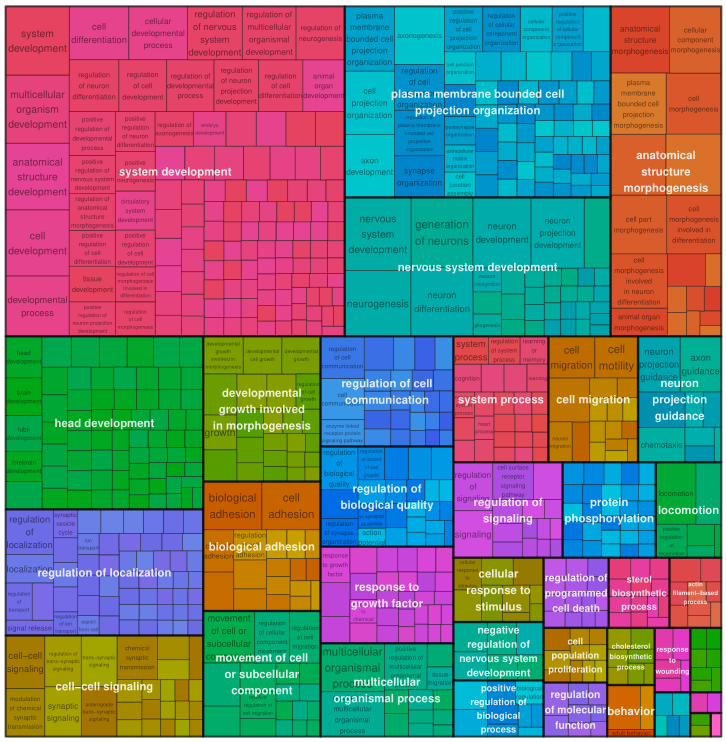

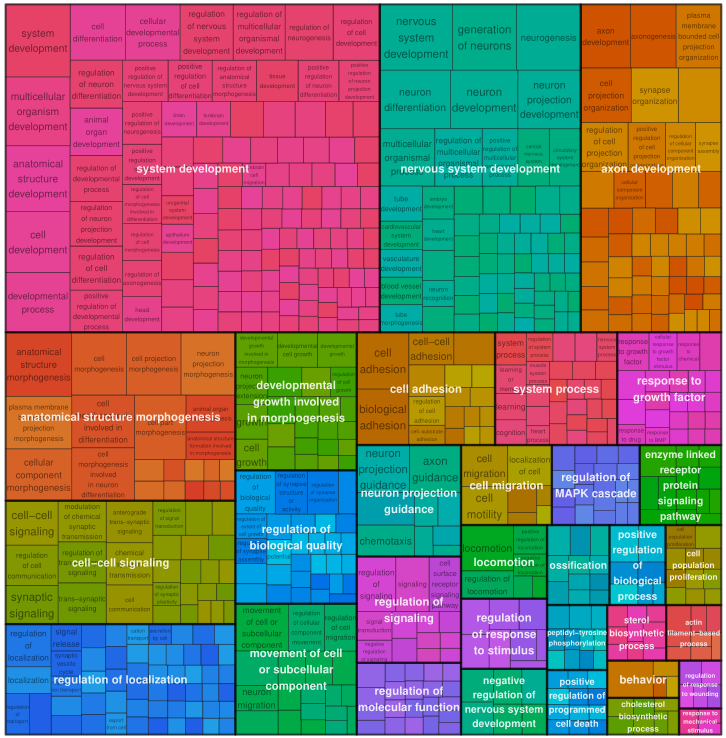


A

B

Figs. S17 Treemap of GO results for DE_CUL7 (A) and DE_DCC (B). Each colored grid represents a cluster of similar GO terms.


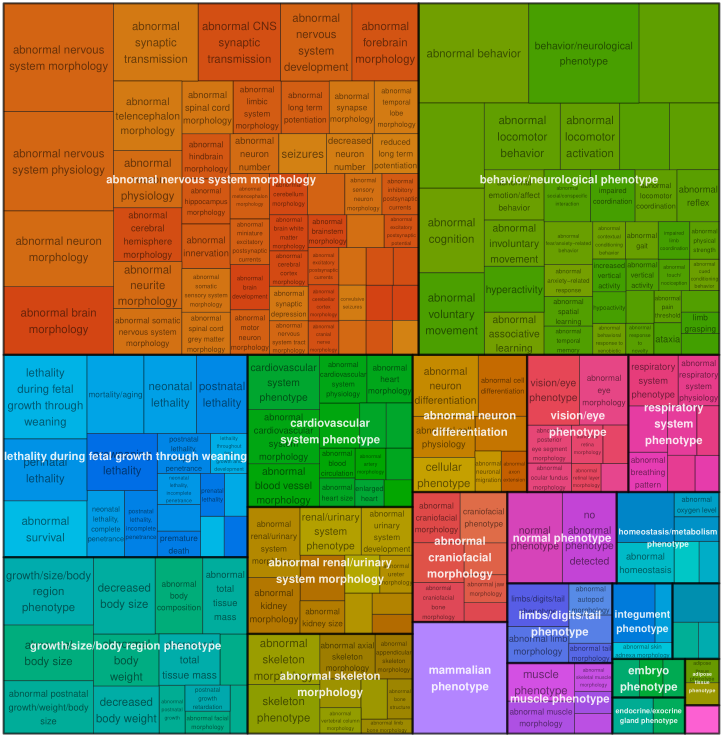

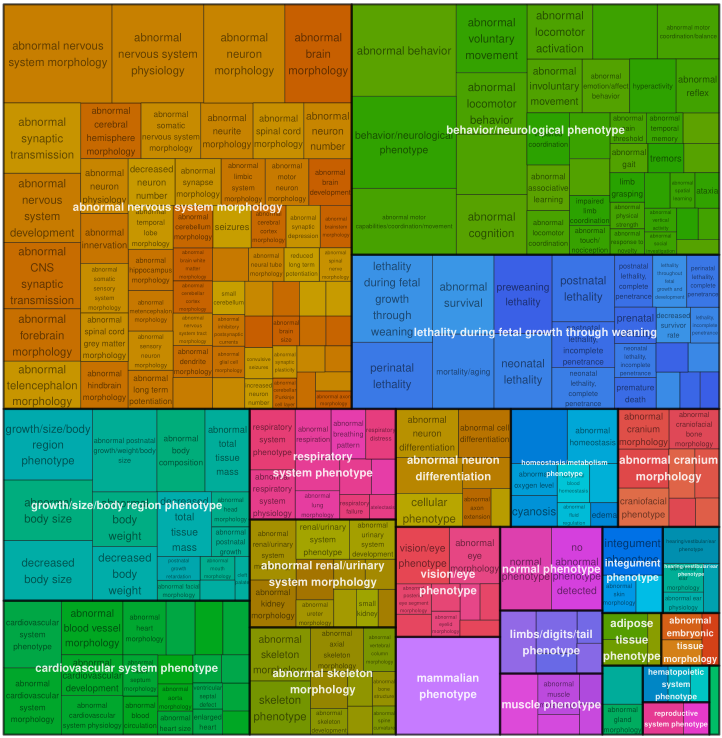


A

B

Figs. S18 Treemap of MPO results for DE_CUL7 (A) and DE_DCC (B). Each colored grid represents a cluster of similar MPO terms.


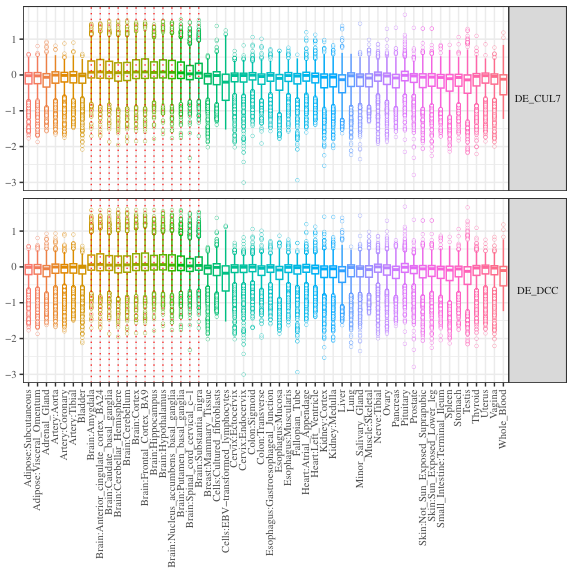


Figs. S19 Expression of DE_CUL7, DE_DCC in GTEx. The DE genes show strong brain specificity.


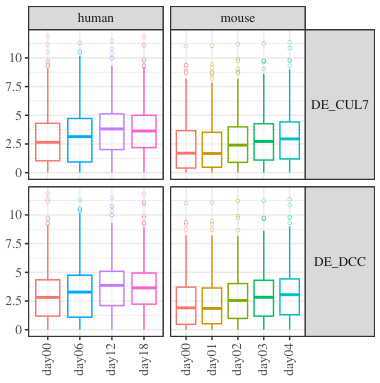


Figs. S20 Expression of DE_CUL7, DE_DCC in developmental neurons of human and mouse. The data is from FANTOM5. The DE genes show elevated expression along with the neuron development.

B

A


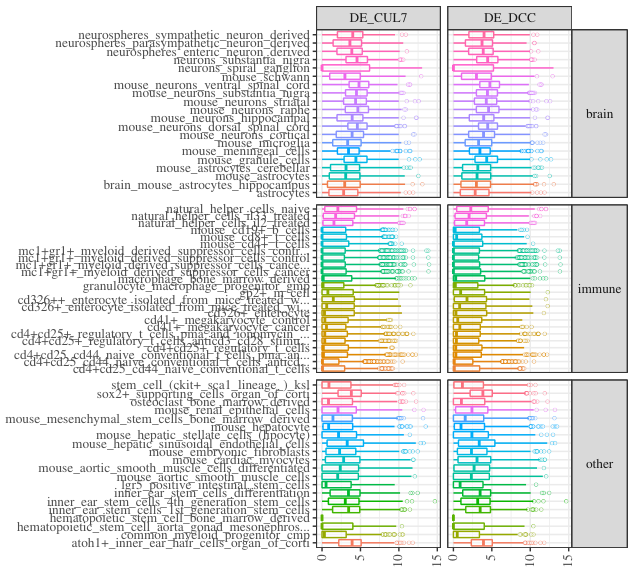

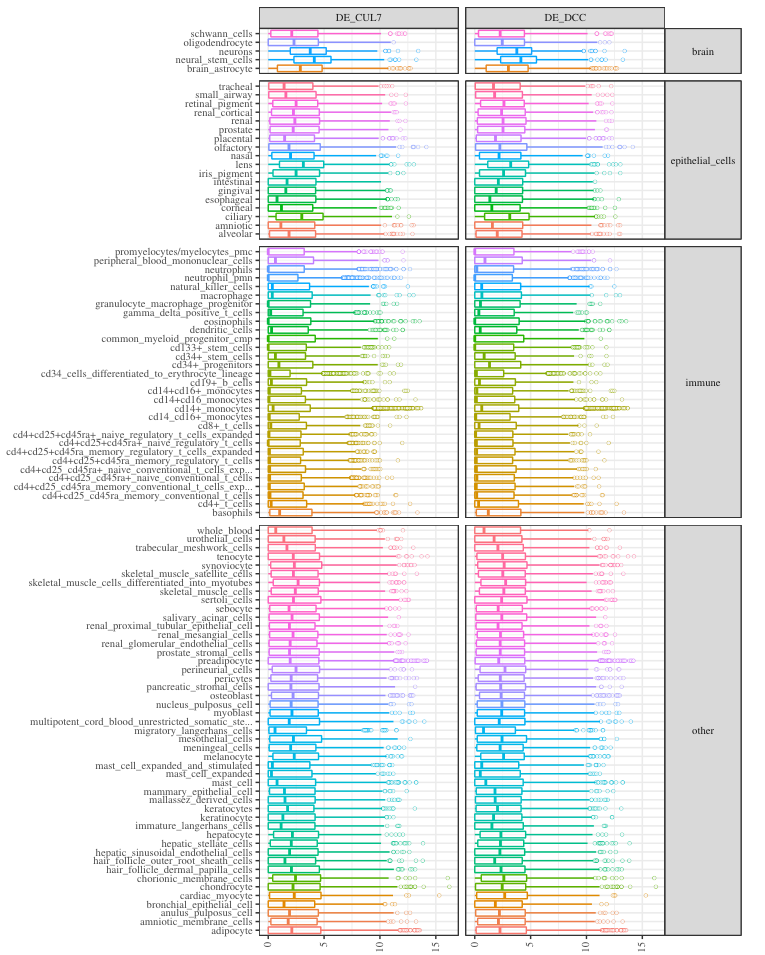


Figs. S21 Expression of DE_CUL7, DE_DCC in primary cell types of mouse (A) and human (B). The data is from FANTOM5. The DE genes show brain cell specificity.


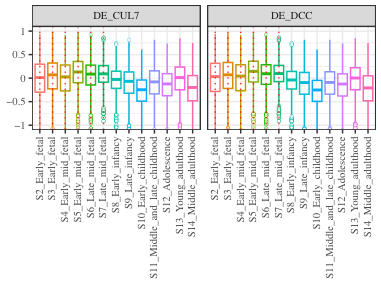


Figs. S22 Expression of DE_CUL7, DE_DCC in the developmental brain of BrainSpan. The DE genes show clear higher expression in fetal stages.

B

A


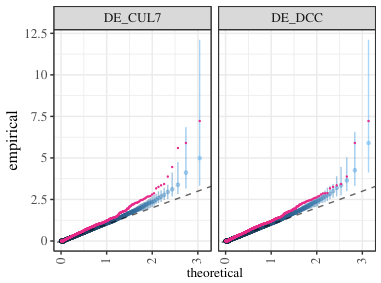

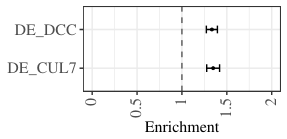


Figs. S23 Genetic evidence of DE_CUL7, DE_DCC. (A) LDSC show the DE gene lists enriched SCZ risk. (B) The DE genes show smaller p values than random gene sets.


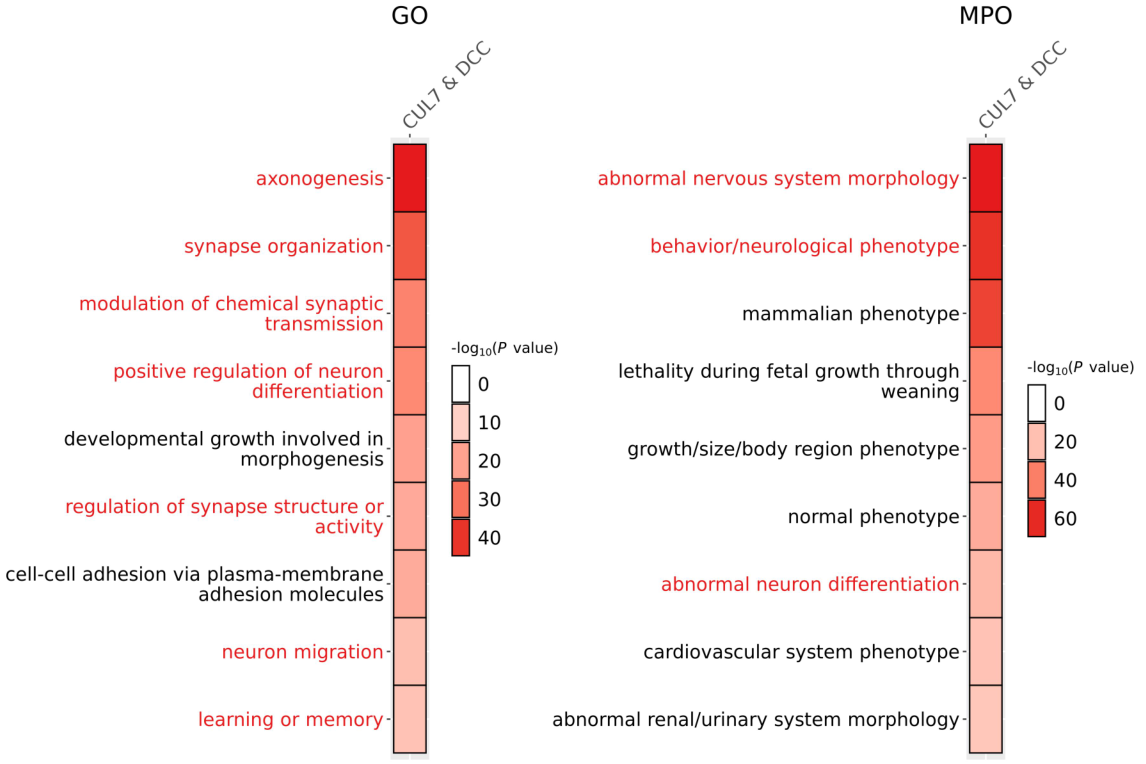


A

B

Figs. S24 Top 15 enriched GO (A) and MPO (B) clusters for overlapped genes of CUL7 & DCC. Red color terms: neuron related term clusters.
